# CK1α protects WAVE from degradation to regulate cell shape and motility in the immune response

**DOI:** 10.1242/jcs.258891

**Published:** 2021-12-09

**Authors:** Alexander Hirschhäuser, Marianne van Cann, Sven Bogdan

**Affiliations:** 1 Institute of Physiology and Pathophysiology, Dept. of Molecular Cell Physiology, Philipps-University Marburg, 35037 Marburg, Germany; 2 Institute for Neurobiology, University of Münster, 48149 Münster, Germany

**Keywords:** *Drosophila*, Macrophages, Cell migration, Cell shape, Lamellipodia, Cell motility, Actin, Arp2/3, WAVE, CK1α, CK2, Phosphorylation, Ubiquitin-dependent protein degradation

## Abstract

The WAVE regulatory complex (WRC) is the main activator of the Arp2/3 complex, promoting lamellipodial protrusions in migrating cells. The WRC is basally inactive but can be activated by Rac1 and phospholipids, and through phosphorylation. However, the *in vivo* relevance of the phosphorylation of WAVE proteins remains largely unknown. Here, we identified casein kinase I alpha (CK1α) as a regulator of WAVE, thereby controlling cell shape and cell motility in *Drosophila* macrophages. CK1α binds and phosphorylates WAVE *in vitro*. Phosphorylation of WAVE by CK1α appears not to be required for activation but, rather, regulates its stability. Pharmacologic inhibition of CK1α promotes ubiquitin-dependent degradation of WAVE. Consistently, loss of *Ck1*α but not *ck2* function phenocopies the depletion of WAVE. Phosphorylation-deficient mutations in the CK1α consensus sequences within the VCA domain of WAVE can neither rescue mutant lethality nor lamellipodium defects. By contrast, phosphomimetic mutations rescue all cellular and developmental defects. Finally, RNAi-mediated suppression of 26S proteasome or E3 ligase complexes substantially rescues lamellipodia defects in CK1α-depleted macrophages. Therefore, we conclude that basal phosphorylation of WAVE by CK1α protects it from premature ubiquitin-dependent degradation, thus promoting WAVE function *in vivo*.

This article has an associated First Person interview with the first author of the paper.

## Abstract (precis)

**Summary:** We identified CK1α as a novel regulator of WAVE controlling cell shape and motility in immune response. Basal phosphorylation of WAVE by CK1α protects it from premature proteasomal degradation.

## INTRODUCTION

Cell shape changes require dynamic remodeling of the actin cytoskeleton. The WASP family verprolin homologous protein (WAVE) is a central Arp2/3 regulator driving lamellipodial protrusions and cell migration in most eukaryotic cells. Together with Abi, NCKAP1/Nap1, CYFIP/Sra-1 and BRK1/HSPC300, WAVE forms a conserved hetero-pentameric complex, the WAVE regulatory complex (WRC). Within the WRC, activity of WAVE towards the Arp2/3 complex is inhibited by intracomplex sequestration of its Arp2/3 activating domain, i.e. the verprolin homology, cofilin homology, acidic region (VCA) domain. One of the central WRC activators is the small RhoGTPase Rac1, which directly binds to the WRC subunit Sra-1 and activates the WRC by allosterically releasing the bound Arp2/3-activating VCA domain of WAVE. Several studies have shown that phosphorylation plays an important role in regulating WRC-Arp2/3-mediated actin filament branching and lamellipodia formation (Mendoza, 2013). WAVE proteins are phosphorylated at numerous sites, and several kinases have been identified as potential regulators (Mendoza, 2013). A previous *in vitro* study has identified multiple functional phosphorylation events within the acidic VCA domain of mammalian WAVE2 (officially known as WASF2) by casein kinase 2 (CK2), which are required for its activity (Pocha and Cory, 2009). However, the results are based on the overexpression of phosphorylation-deficient mutants in cultured NIH-3T3 cells in the presence of the endogenous wild type protein (Pocha and Cory, 2009). A more recent *in vivo* study confirmed that the C-terminal acidic region within the VCA domain of the *Dictyostelium* WAVE is basally phosphorylated at four phosphorylation sites by CK2 and suggested that a regulated dephosphorylation of a fraction of the cellular WAVE pool is a key step in its activation during pseudopod dynamics (Ura et al., 2012).

In this work, we analyze the role of phosphorylation of WAVE (also known as SCAR) in *Drosophila in vivo*. By using RNA interference (RNAi), we identified casein kinase 1α (CK1α) but not CK2 as an important regulator of lamellipodia formation and immune cell migration. *Ck1α*-mutant macrophages exhibit a stellate morphology and an altered migratory behavior that phenocopies *wave-*deficient cells. We also found that CK1α can interact physically with WAVE. *Drosophila* WAVE contains two conserved CK1α consensus sequences that are located in the N-terminal WHD and the C-terminal acidic domain, overlapping with two conserved CK2 phosphorylation sites within mammalian WAVE2. Phosphorylation-deficient mutations in the N-terminal but not C-terminal domain of WAVE can fully rescue the lethality of the *wave* mutant and the lamellipodium defects of macrophages deficient for *wave*. Loss-of and gain-of-function analysis, and pharmacological inhibition of CK1α further suggest that basal phosphorylation of VCA domain by CK1α is crucial for WAVE stability rather than its activity *in vivo*.

## RESULTS

### Loss of *Ck1*α function results in a prominent stellate cell morphology

As previously shown, suppression of Arp2/3-mediated actin polymerization in *arp2*- or *wave*-depleted macrophages results in complete loss of lamellipodial protrusions (Rogers et al., 2003; Zobel and Bogdan, 2013). To screen systematically for candidate protein kinases that are required for lamellipodia formation, we used *Drosophila* macrophages as a model system, therefore combining many advantages of cultured cells with a genetic *in vivo* model system (Rüder et al., 2018; Sander et al., 2013). We tested 308 conditional transgenic RNAi fly lines targeting 162 kinases encoded in the fly genome (see Table S1). Transgene RNAis were specifically coexpressed with GFP in the macrophage lineage using the *hml*Δ-Gal4 driver (Sinenko and Mathey-Prevot, 2004). Macrophages were isolated from third-instar larvae, and tested for their phenotypic effects on lamellipodia formation and cell spreading *ex vivo*.

**Fig. 1.**
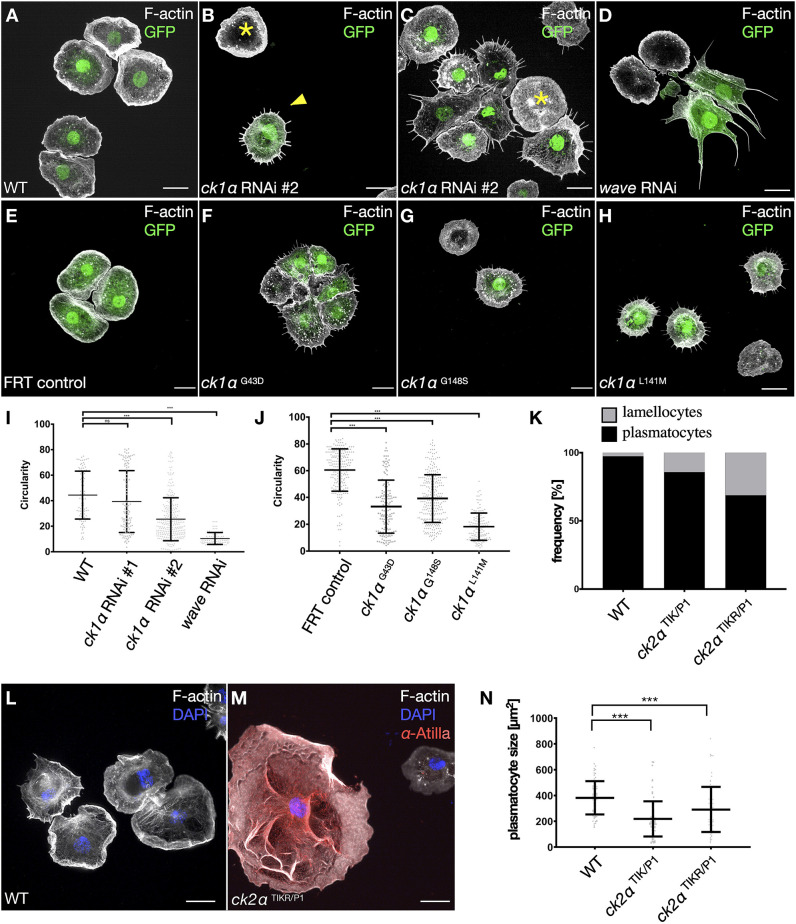
**Loss of CK1α functions disrupt lamellipodia formation.** (A-H) Maximum intensity projection of confocal images that show larval macrophages expressing GFP (green); Alexa Fluor 568-labeled phalloidin was used to stain the actin cytoskeleton (white). Scale bars: 10 µm. (A) Wild type macrophages show a highly polarized actin cytoskeleton with a broad lamellipodial cell front. (B,C) Macrophage-specific knockdown of CK1α in larval macrophages using the *hemolectin*-Gal4 driver disrupts lamellipodia formation. Embryo-derived macrophages that do not co-express *Ck1α* dsRNA and GFP show wild type cell morphology (asterisk). (D) Macrophage-specific knockdown of *wave* results in a complete loss of lamellipodial protrusions. (E) MARCM control clones show a wild type cell morphology. (F-H) *Ck1α*-mutant cells show a stellate cell morphology. (I) Quantification of cell circularity. Macrophage-specific knockdown of *Ck1α* Wild type (WT; *n*=99), *Ck1α* RNAi #1 (*n*=116), *Ck1α* RNAi #2 (*n*=127) and *wave* RNAi (*n*=89), depicted in a scatter dot plot with bars indicating mean±s.d. ***P≤0.001 ANOVA. (J) Quantification of cell circularity. FRT 19 control (*n*=205), *Ck1α* ^G43D^ (*n*=152), *Ck1α* ^G148S^ (*n*=282) and *Ck1α* ^L141M^ (*n*=135) cells, depicted in a scatter dot blot with bars indicating mean±s.d. ***P≤0.001 (one-ANOVA with Dunnett's multiple comparison test). I and J show a unitless measure. (L,M) Structured illumination microscopy (SIM) images of lamellocytes isolated from *Drosophila* larvae stained for Atilla (red) and F-actin (white); nuclei were stained with DAPI (blue). Scale bars: 10 µm. Wild type cells (L), transheterozygous *ck2*α*^TIKR/P1^* mutant cells (M). (K,N) Quantification of lamellocyte frequency (K) and macrophage size (N). Note that loss of *ck2*α induces not only lamellocytes at the expense of plasmatocytes but also results in macrophages with reduced cells size, likely to represent intermediates.

Wild type macrophages acquire a round, pancake-like shape with a broad lamellipodial actin filament network (Fig. 1A). Expression of most double-stranded RNAs (dsRNAs) induced no defects in the cell morphology and lamellipodia formation (see Table S1). We identified the casein kinase 1α (*Ck1α*) gene as a candidate that most strongly affected lamellipodia formation and phenocopied *wave-*depleted cells, characterized by a prominently reduced circularity index (Fig. 1B–D,I). We only found a few more dsRNAs that affected cell shape (see Table S1).

Next, we analyzed *Ck1α* mutants, bearing distinct missense mutations (*Ck1α*^8B12^, *Ck1α*^A^ and *Ck1α*^B^). Of those, *Ck1α*^8B12^ (*Ck1α*^G43D^) is the only functionally characterized *Ck1α* allele carrying a mutation that transforms the conserved glycine residue at position 43 into an aspartic acid (G43D). It has been first described as a strong hypomorph or amorphic allele (Legent et al., 2012). *Ck1α*^A^ (*Ck1α*^L141M^) and *Ck1α*^B^ (*Ck1α*^G148S^) have been previously isolated in a large EMS screen, but neither allele has so far been functionally characterized (Haelterman et al., 2014). *Ck1α*^A^ carries a mutation that leads to replacement of a conserved lysine residue with methionine at position 141 (L141M), constituting the only mutation that yields removal of an H-bond – which might affect the active site of the CK1 (see 3D structure in Movie 1, dashed lines in magenta). By contrast, *Ck1α*^B^ replaces glycine with a serine at position 148 (G148S) without any obvious structural changes.

To analyze these embryonic lethal *Ck1α* mutations in macrophages we performed mosaic analysis with a repressible cell marker (MARCM; Wu and Luo, 2006) to generate *Ck1α*-mutant macrophages in a wild type animal background. Compared with control cells (Fig. 1E), isolated GFP-positive *Ck1α*^L141M^ mutant cells displayed the strongest defects in lamellipodia formation, showing a prominent stellate cell morphology compared with *Ck1α*^G43D^ and *Ck1α*^G148S^ (Fig. 1E–H, quantification of reduced circularity is shown in Fig. 1J). Thus, we confirmed CK1α as an important novel regulator of cell shape.

A possible role on cell shape has also been suggested for casein kinase 2 (CK2; Kramerov et al., 2011; Pocha and Cory, 2009), a structurally completely different enzyme that was also included in our initial RNAi screen (see also Table S1). Whereas CK1α is a monomeric serine kinase, CK2 is composed of two catalytic CK2α and two regulatory CK2β subunits that form a hetero-tetrameric (α2β2) holoenzyme (Bandyopadhyay et al., 2017). RNAi-mediated depletion of either CK2α or CK2β subunit did not significantly affect lamellipodia formation but resulted in altered blood cell homeostasis, which could be also confirmed in *ck2α* (also known as *CkII*α)-mutant larvae (Table S1; Fig. 1K–M). Loss of *ck2α* function induced the formation of Atilla-positive lamellocytes at the expense of macrophages (Fig. 1L,M; quantification in Fig. 1K). Lamellocytes are giant cells that are rarely observed in healthy flies, but transdifferentiation from macrophages is dramatically induced in response to infection by parasitic wasps (Anderl et al., 2016). Transheterozygous *ck2α*^TIK/P1^ and *ck2α*^TIKR/P1^ mutant larvae, which lack CK2α kinase activity (Bulat et al., 2014), showed an enlarged lamellocyte compartment (≤30%) and macrophages (70%) whose average size differs compared with control macrophages (Fig. 1N). as recently suggested, these cells might be at an intermediate state of transdifferentiation (Anderl et al., 2016). Our data suggest that CK2 regulates blood cell differentiation, rather than blood cell shape.

### Loss of *Ck1*α function impairs macrophage migration and immune response

**Fig. 2.**
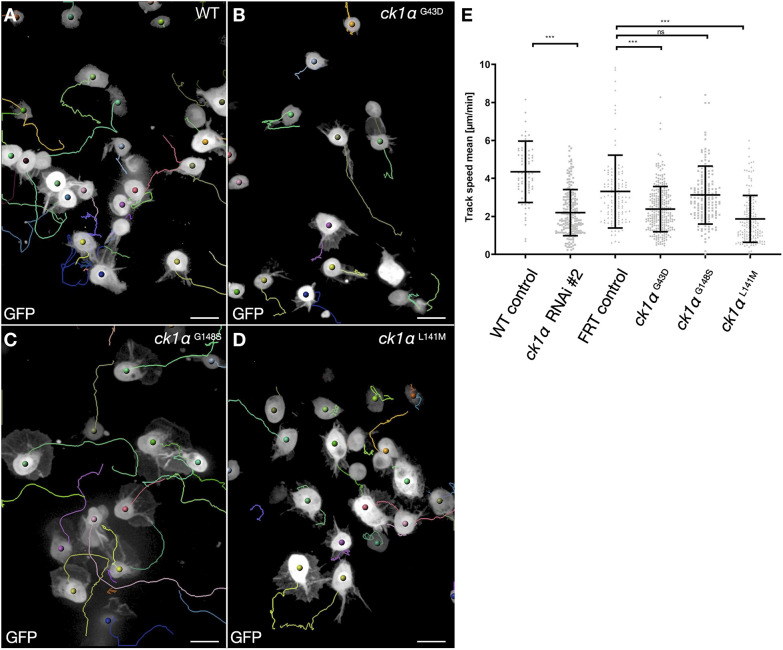
**Loss of CK1α impairs macrophage migration.** (A–D) Still images of randomly migrating pupal macrophages (after 20 min recording). Cells were tracked using Imaris software; single trajectories are depicted in different colors. Wild type macrophages (A) expressing GFP show broad lamellipodia that migrate constantly. Mutation of *Ck1α* (B–D) results in reduced cell speed. Scale bars: 10 µm. (E) Quantification of cell speed. Shown is a scatter dot plot with bars indicating mean±s.d. ***P≤0.001 (Welch's *t*-test); ns, not significant. The mean track speed of *Ck1α ^L141M^>Ck1α ^G43D^* mutant cells is significantly reduced comparison to control macrophages. Based on lamellipodium defects and impaired migratory behavior, we ranked the mutations into the following allelic series: *Ck1α* ^L141M^>*Ck1α* ^G43D^>*Ck1α* ^G148S^.

To further examine a possible role of CK1α in cell migration, we first analyzed the migratory behavior of *Ck1α*^L141M^ mutant macrophages expressing GFP in pre-pupae. Wild type control macrophages form broad lamellipods to migrate along the epidermis (Fig. 2A). Cell trajectories show wild type macrophages migrate long distances within 20 min of acquisition (Fig. 2A; Movie 2; quantification of migration speed is shown in Fig. 2E). By contrast, *Ck1α*^G43D^ and *Ck1α*^L141M^ mutant macrophages migrate considerably slower (Fig. 2B,C,E). No significant differences were observed between (FRT, where FRT indicates the target site for the FLP recombinase) control and *Ck1α*^G148S^ mutant macrophages, suggesting that *Ck1α*^G148S^ is a hypomorph (Fig. 2D,E).

**Fig. 3.**
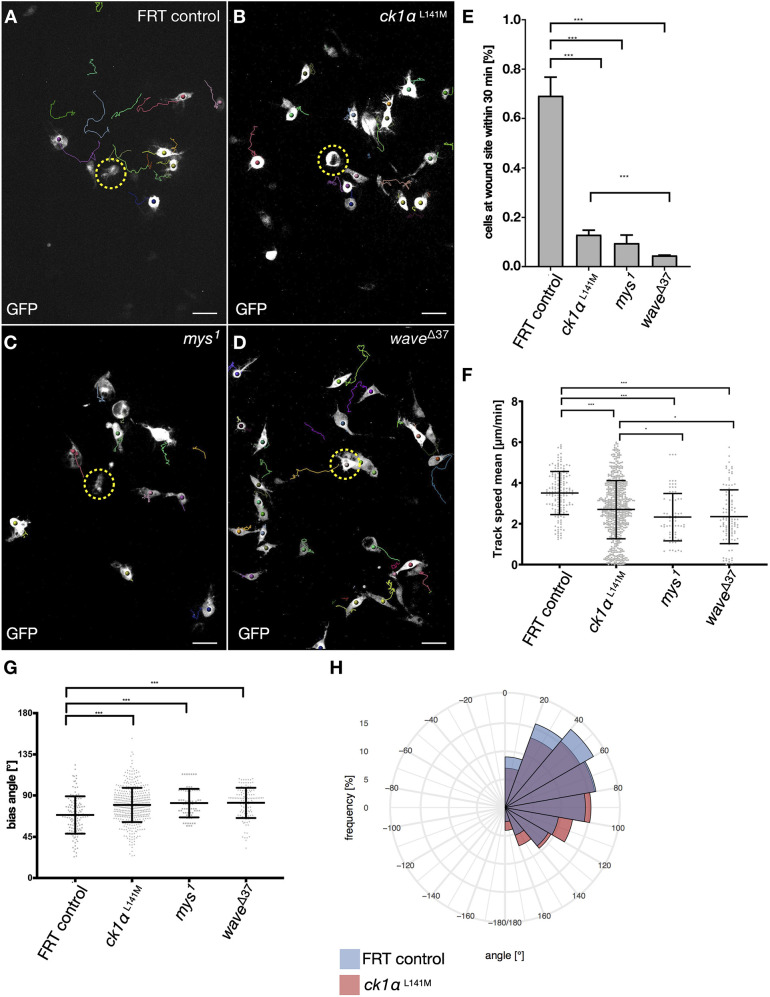
**Loss of CK1α impairs macrophage directionality upon wounding of a single cell.** (A–D) Still images of spinning disc time-lapse movies of directed macrophage migration upon wounding of a single cell (encircled by yellow dashed line, *t*=0). Scale bars: 20 μm. Cells were imaged for 30 min after laser ablation in 30 s intervals and tracked by using Imaris software. (A) Upon wounding, wild type cells switch from random to directed migration. Within 30 min, most cells within a distinct radius reach the wound site. (B) Trajectories indicate that *Ck1α-*mutant macrophages are attracted to the wound site, but they migrate with a reduced directionality. A similar, but even stronger impaired migratory behavior was found for *mys^1^* (C) and *wave*^Δ37^ (D) mutant macrophages. (E) Quantification of cells at the wounding site 30 min after laser ablation of wild type FRT19 control, *Ck1α*^L141M^, *mys^1^* and *wave*^Δ37^ cells. ***P≤0.001 (Welch's *t*-test). (F) Quantification of cell speed. Shown is a scatter dot plot, with bars indicating mean±s.d. ***P≤0.001, *P≤0.033 (Welch's *t*-test). The mean track speed of *mys* and *wave*^Δ37^>*Ck1α^L141M^* mutant cells is significantly reduced compared to that of control macrophages. Wild type FRT19 control (*n*=130), *Ck1α*^L141M^ (*n*=445), *mys^1^* (*n*=72) and *wave*^Δ37^ (*n*=120). (G) Quantification of the bias angle. Wild type FRT19 control (*n*=130), *Ck1α*^L141M^ (*n*=445), *β^PS^-integrin* (*mys*, *n*=72) and *wave*^Δ37^ (*n*=120). Shown is a scatter dot blot with bars indicating mean±s.d. ***P≤0.001 (Welch's *t*-test). (H) Polar histogram chart of the bias angle distribution, showing impaired directionality of *Ck1α*^L141M^ mutant macrophages when compared to wild type FRT control cells.

To determine the role of CK1α in directed wound response of macrophages, laser ablation experiments were performed in a single cell within the pupal wing (Fig. 3A–D, area encircled by dashed yellow line). Upon wounding, control macrophages (FRT control) switch from random to directed migration towards the wounding site. Cells were automatically tracked within the first 30 min post wounding and trajectories were constructed (Fig. 3A–D; quantification in Fig. 3E–H; Movie 3). *Ck1α*-deficient macrophages still respond to the cell damage, however, due to reduced lamellipodia formation, *Ck1α*^L141M^ mutant cells were impaired in their ability to migrate towards the ablated cell. To better characterize defects in the migratory behavior we first counted the number of cells that reached the wound within the first 30 min after wounding (Fig. 3E). Number of cells at the wound were normalized to the total number of cells within 10–80 µm of the ablation site. We also measured the mean track speed of mutant cells compared to that of FRT control cells (Fig. 3F). Both, cell numbers at the wound and cell speed were significantly reduced in *Ck1α*^L141M^ as well as in *myospheroid* (*mys*^1^) and *wave*^Δ37^ mutant cells (Fig. 3E,F; Movie 3). To further describe the impaired migratory behavior of *Ck1α*^L141M^ mutant cells we also measured the bias angle, i.e. the angle between the motion vector (a step of the cell) and the direction vector pointing towards the wound (Liepe et al., 2016; Weavers et al., 2016). A bias angle of ∼0° indicates the highest directionality to the wound, whereas cells with a value of ∼180° move in the opposite direction. For migrating wild type macrophages, the bias angle had values of <80° (shown in Fig. 3G,H as frequency distribution of the bias angle for each trajectory). By contrast, *Ck1α*^L141M^ mutant cells move with a bias angle between 80° and 180° (Fig. 3G,H). *Ck1α-*mutant macrophages showed reduced distance from the origin, with a reduced directionality (indicated by the cell bias angle; Fig. 3F). A similar, but more severely impaired migratory behavior was shown for *wave^Δ37^* and β-integrin (*mys*^1^) mutant macrophages (Fig. 3F,G; Moreira et al., 2013). These results indicate that CK1α is required for proper lamellipodia formation and immune cell migration *in vivo*.

### CK1α physically interacts with WAVE and human recombinant CK1δ can phosphorylate *Drosophila* WAVE *in vitro*

**Fig. 4.**
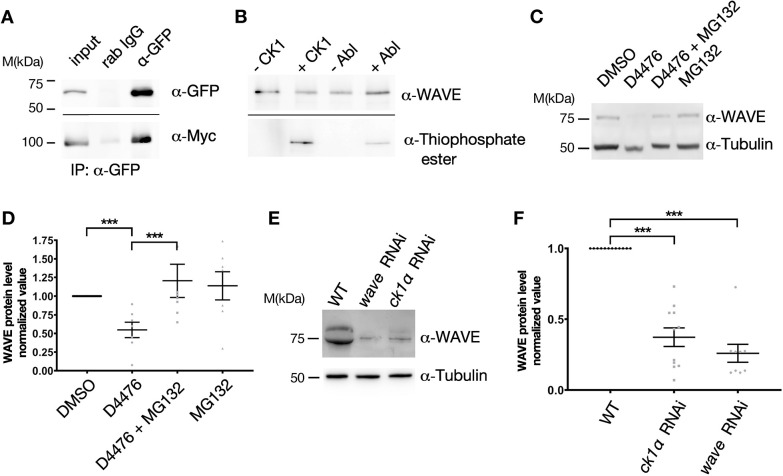
**RNAi-induced depletion of CK1α results in reduction of WAVE protein levels in macrophages.** (A) CK1α physically interacts with WAVE. S2 cells were co-transfected with EGFP-tagged CK1α and Myc-tagged WAVE. Total cell lysates (input) were used for immunoprecipitation with a pre-immune control serum [rabbit (rab) IgG] or α-GFP serum (α-GFP). Samples were separated using SDS-PAGE and analyzed by western blotting using antibodies against GFP (α-GFP, top) or Myc (α-Myc, bottom). (B) Detection of *in vitro* phosphorylated WAVE protein, phosphorylated through recombinant human CK1 or Abl. Reactions were performed using recombinant GST-WAVE purified from *E. coli*. −/+ indicates absence (−) and presence (+) of the CK1 or Abl (NEB). Reaction conditions depend on the added kinase. Samples were separated by SDS-PAGE and analyzed by western blotting using anti-WAVE and anti-thiophosphate ester antibodies (α-WAVE and α-thiophosphate ester, respectively). (C) Inhibition of CK1α increases WAVE protein levels. S2 cells were treated with vehicle control (DMSO), the CK1α-specific inhibitor D4476, the proteasome inhibitor MG132 or D4476 and MG132 together for 4 h. The cells were collected, lysed and analyzed by immunoblotting with antibodies against WAVE or tubulin. Inhibition of CK1α decreases levels of endogenous WAVE protein, whereas MG132-treated cells show markedly increased levels. Tubulin served as loading control. (D) Quantification of WAVE protein levels in response to pharmacological inhibition of CK1α. Results are the average of five independent experiments normalized to wild type protein level (set at 1). *P*-values from ratio paired *t*-test are shown when significantly different from control (***P<0.001). (E) RNAi induced CK1*α* depletion results in substantial reduction of WAVE protein levels in macrophages. Larval macrophages were isolated, centrifuged and resuspend in SDS-sample buffer, and immunoblotted with antibodies against WAVE or tubulin. RNAi of *wave* serves as control to clarify specificity and reduction of WAVE protein levels. Tubulin serves as the loading control. (F) Quantification of WAVE protein levels upon RNAi-mediated knockdown. RNAi of *wave* serves as control. Results are the average of nine independent experiments. *P*-values from ratio paired *t*-test are shown when significantly different from control (***P<0.001).

Given the similar cellular phenotypes, we next tested for a possible physical interaction between CK1α and WAVE. To this end, we transiently co-transfected *Drosophila* S2R+ cells with EGFP-tagged CK1α and Myc-tagged WAVE, followed by coimmunoprecipitation experiments. Pull-down assays using lysates from cells expressing tagged CK1α and WAVE revealed a physical interaction between the two proteins (Fig. 4A). To further examine whether CK1 can phosphorylate WAVE, we performed an *in vitro* kinase assay using recombinant human CK1δ kinase (1–317aa) – which has 65% identity to *Drosophila* CK1α – in the presence of purified glutathione S-transferase (GST)-tagged *Drosophila* WAVE protein (GST-WAVE). In this assay, ATPγS served as the phosphate donor from which mono-thiophosphate instead of phosphate is transferred to the substrate (Fig. 4B). Alkylated thiophosphate creates an epitope for thiophosphate ester-specific antibodies, which allows detection of phosphorylation (Allen et al., 2007). Our thiophosphorylation assay showed that WAVE can be phosphorylated by CK1 and its positive control Abelson kinase (Abl) (Leng et al., 2005) (Fig. 4B).

### CK1α protects WAVE from ubiquitin-mediated degradation

**Fig. 5.**
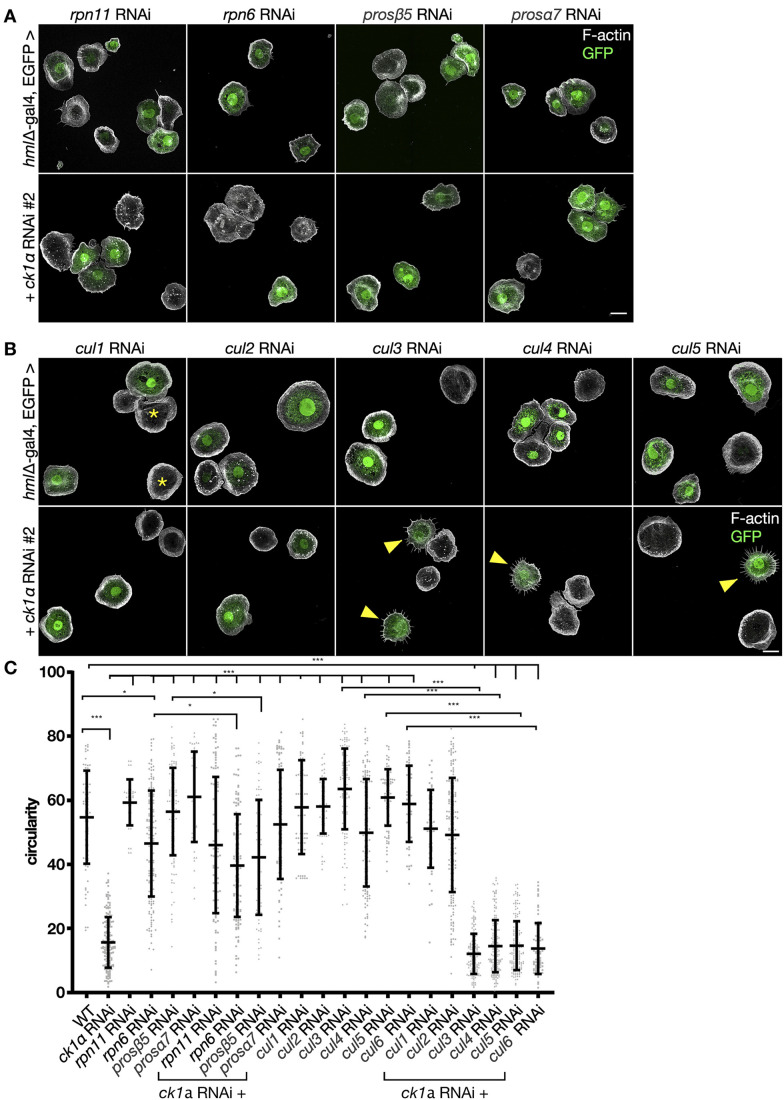
**Lamellipodia defects in CK1α-depleted macrophages are rescued upon inhibition of proteasomal degradation.** (A,B) Maximum intensity projection of confocal images that show larval macrophages expressing GFP (green). Alexa Fluor 568-labeled phalloidin was used to stain the actin cytoskeleton (white). Scale bar: 10 µm. (A) Macrophage-specific knockdown of proteasome components *rpn11*, *rpn6*, *prosβ5* or *prosα7* in larval macrophages using the *hemolectin*-Gal4 driver does not affect cell morphology. Rescue of cell morphology defects of *Ck1α* RNAi-depleted cells by co-expression of indicated transgenic RNAis against indicated proteasomal components. (B) Macrophage-specific knockdown of any of the six known cullin proteins does not significant impact on cell shape. Embryo-derived macrophages that do not co-express dsRNA and GFP are indicated (asterisks). RNAi-mediated knockdown of Cul1 and Cul2 but not Cul3 to Cul6 rescued cell shape defects of macrophages evoked by *Ck1α* RNAi. Scale bars: 10 µm. (C) Quantification of cell circularity. Shown is a scatter dot plot, bars indicate mean±s.d. *P≤0.033, ***P≤0.001 (one-way ANOVA with Bonferroni's multiple comparison test). Wild type WT (*n*=78) *Ck1α* RNAi (*n*=172), *rpn11* RNAi (*n*=40), *Rpn6* RNAi (*n*=163), *prosβ5* RNAi (*n*=117), *prosα7* RNAi (*n*=47), *Ck1α* RNAi+r*pn11* RNAi (*n*=124), *Ck1α* RNAi+*rpn6* RNAi (*n*=145), *Ck1α* RNAi+*prosβ5* RNAi (*n*=74) and *Ck1α* RNAi+*prosα7* RNAi (*n*=83), *Cullin 1* RNAi (*n*=61), *Cullin 2* RNAi (*n*=66), *Cullin 3* RNAi (*n*=120), *Cullin 4* RNAi (*n*=130), *Cullin 5* RNAi (*n*=90), *Cullin 6* RNAi (*n*=54), *Ck1α* RNAi+*Cullin 1* RNAi (*n*=48), *Ck1α* RNAi+*Cullin 2* RNAi (*n*=174), *Ck1α* RNAi+*Cullin 3* RNAi (*n*=144), *Ck1α* RNAi+*Cullin 4* RNAi (*n*=177), *Ck1α* RNAi+*Cullin 5* RNAi (*n*=170), *Ck1α* RNAi+*Cullin 6* RNAi (*n*=76).

We next analyzed the potential effect of CK1α on endogenous WAVE protein. First, we used the cell-permeable, CK1α-specific ATP-competitive inhibitor D4476 (Rena et al., 2004). Remarkably, inhibition of CK1α activity by D4476 resulted in a significant reduction of WAVE protein levels in S2 cells (Fig. 4C, quantification is shown in Fig. 4D). This was prevented by addition of the proteasome inhibitor MG132, suggesting that phosphorylation of WAVE by CK1α protects WAVE from ubiquitin-mediated degradation (Fig. 4C,D). In addition, we found that CK1α depletion through RNAi results in substantial reduction of WAVE protein levels in larval macrophages (Fig. 4E). Quantification of nine independent experiments is shown in Fig. 4F. To further analyze whether inhibition of ubiquitin-mediated degradation can also revert the lamellipodium defects evoked by depletion of CK1α, we screened for proteasome components and tested various RNAi lines. Ubiquitin-dependent degradation is a multi-step process that involves members of the cullin protein family as part of E3 ligase complexes (Morreale and Walden, 2016) as well as the 26S proteasome, consisting of a 20S catalytic core and a 19S regulatory complex (Saeki, 2017). Upon inhibition of proteasomal degradation by targeting either the 20S catalytic core (Prosβ5, Prosβ7) or the 19S regulatory complex (Rpn11, Rpn6), we found that the lamellipodium defects induced in response to RNAi of *Ck1α* were significantly rescued, whereas knockdown of *rpn11*, *rpn6*, *prosβ5* and *prosβ7* alone did not show significant differences regarding cell shape (Fig. 5A,C). We also tested the six known *Drosophila* members of the cullin protein family (Cul1, 2, 3, 4, 5 and 6), which function as scaffolds to assemble distinct E3 ligase complexes (Ketosugbo et al., 2017). Interestingly, RNAi-mediated knockdown of Cul1 and Cul2, but not Cul3 to Cul6 (RNAi had already been validated), rescued cell shape defects of macrophages evoked by RNAi of *Ck1α* (Fig. 5B,C). Knockdown of any cullin protein alone did not show significant differences in cell shape (Fig. 5B,C). Taken together, these data further provide evidence that CK1α protects WAVE from ubiquitin-mediated proteasomal degradation to ensure the proper formation of lamellipodia.

We also tested whether forced overexpression of CK1α might increase WAVE protein levels. For this, we generated a stably transfected S2 cell line expressing CK1α under the control of a Cu^2+^-inducible metallothionein promoter (pMT). However, induced expression of full-length CK1α did neither induce a phosphorylation-dependent mobility shift of endogenous WAVE nor did it yield increased levels of WAVE protein (Fig. S1A,B), suggesting that prominent basal phosphorylation by CK1α already stabilizes endogenous WAVE levels.

### Phosphorylation of the acidic region within the VCA domain of WAVE is essential for its function regarding lamellipodia formation, cell migration and development

**Fig. 6.**
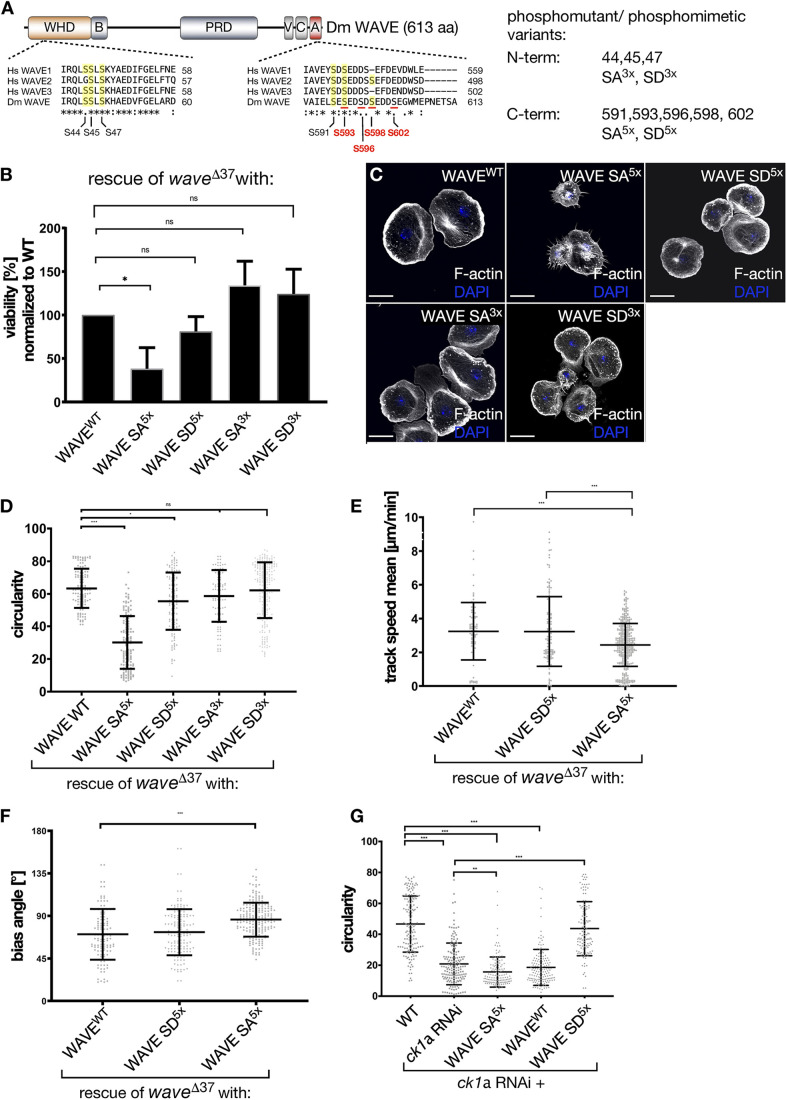
**Phosphorylation of the acidic region within the VCA domain of WAVE is essential for its function.** (A) Schematic of domain structure within the *Drosophila* WAVE protein. Shown are the WAVE homology domain (WHD), basic region – B, proline-rich region – PRG, verprolin domain – V, central region – C and acidic region – A, as well as sequence alignment of N-terminal and C-terminal parts of human of WAVE1, 2 and 3, and *Drosophila* WAVE. Shown are two CK1α consensus sequences within the WHD and the acidic region of WAVE; conserved serine residues are underlined in yellow. Serine residues that match the CK1α consensus motif are underlined and/or shown in red. Phosphorylatable serine residues that were replaced with alanine in the unphosphorylatable mutant (SA) and with aspartic acid in the phosphomimetic mutant (SD) are indicated on the right. (B) Rescue of *wave^Δ37^* homozygote lethality into adulthood by ubiquitous re-expression of transgenic WAVE variants as indicated. Results show the averages of three independent crossings. Notice that only the C-terminal phosphomutant SA^5x^ variant is unable to rescue lethality. *P≤0.03, ns, not significant (one-way ANOVA with Dunnett's multiple comparison test). (C) Maximum intensity projection of confocal images that show larval *wave^Δ37^* mutant macrophages re-expressing transgenic WAVE variants as indicated. Cells were stained for F-actin (white); nuclei were stained with DAPI (blue). Scale bars: 10 µm. N-terminal (SD^3x^) and C-terminal phosphomimetic (SD^5x^) as well as the N-terminal unphosphorylatable mutant (SA^3x^) can substantially rescue the *wave*-mutant phenotype. In contrast, lamellipodia formation is still severely defective in macrophages re-expressing the C-terminal unphosphorylatable mutant of the VCA domain (SA^5x^). (D) Quantification of cell circularity of rescued macrophages. WAVE-WT (*n*=122), WAVE-SA^5x^ (*n*=143), WAVE-SD^5x^ (*n*=131), WAVE-SA^3x^ (*n*=92), WAVE-SD^3x^ (*n*=246). Shown is a scatter dot blot with bars indicating mean±s.d. *P=0.010, ***P≤0.001 ANOVA; ns, not significant. (E) Quantification of mean track speed of directed migration of rescued macrophages upon wounding of a single cell. WAVE-WT (*n*=94), WAVE-SA^5x^ (*n*=384), WAVE-SD^5x^ (*n*=128). Shown is a scatter dot plot with bars indicating mean±s.d. ***P≤0.001 (Welch's *t*-test). (F) Quantification of bias angles of directed migration of rescued macrophages upon wounding of a single cell. WAVE-WT (*n*=296), WAVE-SA^5x^ (*n*=206), WAVE-SD^5x^ (*n*=233). Shown is a scatter dot blot with bars indicating mean±s.d. ***P≤0.001 (Welch's *t*-test). (G) Quantification of cell circularity. Rescue of cell morphology defects of *Ck1α* RNAi-depleted cells by co-expression of indicated transgenic WAVE variants. D and G show a unitless measure. WT (*n*=160), *Ck1α* RNAi (*n*=160), *Ck1α* RNAi+WAVE-SA^5x^ (*n*=131), *Ck1α* RNAi+WAVE-WT (*n*=154) and *Ck1α* RNAi+WAVE-SD^5x^ (*n*=120). Graph is depicted in a scatter dot plot with bars indicating mean±s.d. **P≤0.01, ***P≤0.001 ANOVA.

CK1α is a monomeric, serine/threonine-specific protein kinase that recognizes the canonical consensus sequence [S(p)/T(p)-X-X-S/T] [where S(p)/T(p) indicates a phosphorylated residue (Flotow et al., 1990) and X represents any amino acid]. In addition, non-canonical consensus sequences recognized by CK1 family members have been described previously, i.e. the SLS motif as found in β-catenin (Marin et al., 2003). WAVE proteins contain a conserved SLS motif in the N-terminal WAVE homology domain (WHD) and a canonical CK1α consensus sequence in the C-terminal acidic region comprising VCA (Fig. 6A, marked in red). To further explore the physiological relevance of phosphorylation, we performed rescue experiments in fly. We generated different mutant WAVE variants, in which the serine residues within the N-terminal WHD (i.e. S44, S45 and S47) or the C-terminal VCA domain (i.e. S591, S593, S596, S598 and S602) were replaced with unphosphorylatable alanine residues yielding SA mutants (N-terminal SA^3x^ and C-terminal SA^5x^ mutant variants) (Fig. 6A) or with phosphomimetic aspartic acid residues yielding SD mutants (i.e. N-terminal SD^3x^- and C-terminal SD^5x^ mutant variants) (Fig. 6A). To ensure equal expression rates we integrated these transgenes into the same landing site (68E) using the ΦC31-mediated transgenesis strategy (Bischof et al., 2007).

Ubiquitous re-expression of N-terminal phosphomutant or phosphomimetic variants (SA^3x^ or SD^3x^) fully rescued embryonic lethality of *wave* mutants, indicating that the SLS motif is dispensable for WAVE function (Fig. 6B). By contrast, phosphorylation of the C-terminal acidic domain of WAVE is crucial for WAVE function. With only a small number of progenies, the phosphomutant variant (SA^5x^) failed to rescue the lethality of the *wave* mutant, whereas the C-terminal phosphomimetic variant (SD^5x^) completely restored viability (Fig. 6B). A similar phenotypic analysis in macrophages further confirmed that basal phosphorylation of the C-terminal acidic domain is required for WAVE function (Fig. 6C). Defects regarding lamellipodia formation in *wave*-mutant macrophages can be substantially rescued by re-expressing wild type, N-terminal phosphomimetic SD^3x^ or phosphomutant SA^3x^ WAVE protein (Fig. 6C). However, C-terminal phosphomutant SA^5x^ WAVE failed to rescue the lamellipodium defects (Fig. 6C). Cells expressing the SA^5x^ variant still showed strongly reduced circularity and mean track speed compared to cells rescued either by wild type, SA^3x^ or SD^5x^ protein (quantification in Fig. 6D and E). Similarly, we still found significant defects in the migratory directionality of mutant macrophages expressing phosphomutant SA^5x^ WAVE compared to wild type or the phosphomimetic SD^5x^ variant (quantification in Fig. 6F). Finally, we tested whether defects in lamellipodia formation evoked by RNAi of *Ck1α* can be rescued by overexpression of the phosphomimetic SD^5x^ variant. Indeed, overexpression of the phosphomimetic SD^5x^ but not the phosphomutant SA^5x^ WAVE variant substantially rescued cell shape defects of macrophages deficient for *Ck1α* (Fig. 6G)*.* Likewise, re-expression of wild type WAVE did not rescue defects evoked by RNAi of *Ck1α*, suggesting that basal phosphorylation of the acidic domain of WAVE is essential for its stability *in vivo*.

### Phosphorylation of the acidic region within the VCA domain of WAVE promotes its stability rather than its actin nucleation *in vivo*

**Fig. 7.**
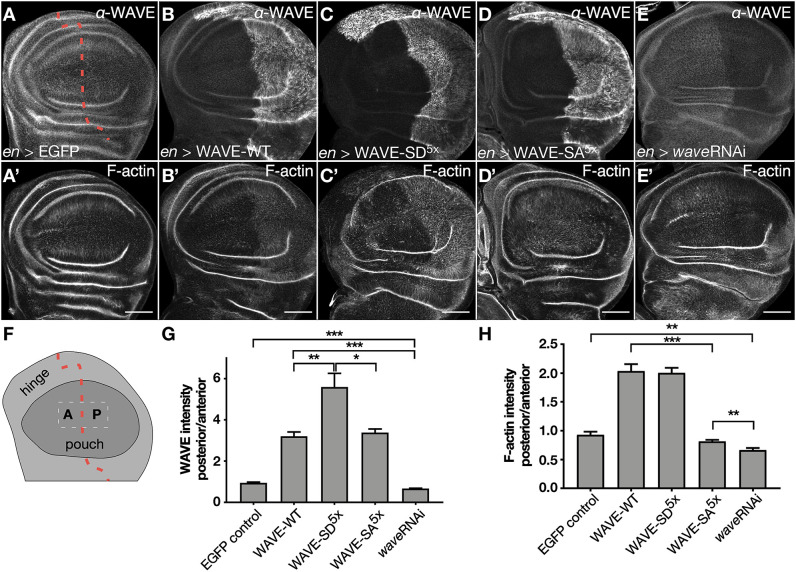
**Phosphorylation of the acidic region within the VCA domain of WAVE promotes its stability rather than its actin nucleation *in vivo.*** (A–E′) Confocal images of wing imaginal discs expressing unphosphorylatable mutant WAVE-SA^5x^ or phosphomimetic WAVE-SD^5x^ constructs in the *en*-Gal4 pattern. Expression of transgenes is verified by antibody staining as indicated. Anterior is to the left. The anterior–posterior compartment border is indicated by a red dashed line. Scale bars: 50 µm. (F) Schematic representation of third-instar larvae wing imaginal disc. A, anterior; P, posterior. The anterior–posterior compartment border is indicated by a red dashed line. (G) Quantification of WAVE levels upon overexpression of WAVE. Quotient of posterior over anterior signal strength. EGFP serves as a negative control and *wave* RNAi transgene as a positive control. WAVE-WT (*n*=15), WAVE-SA^5x^ (*n*=143), WAVE-SD^5x^ (*n*=9), WAVE-SA^3x^ (*n*=14), EGFP (*n*=5), *wave* RNAi (*n*=9). (H) Quantification of F-actin upon overexpression of WAVE as the quotient of posterior/anterior signal intensity. EGFP serves as a negative control and *wave* RNAi transgene as a positive control. *P≤0.033, **P≤0.002, ***P<0.001 (Welch's *t*-test). WAVE-WT (*n*=14), WAVE-SA^5x^ (*n*=143), WAVE-SD^5x^ (*n*=16), WAVE-SA^3x^ (*n*=16), EGFP (*n*=8), *wave* RNAi (*n*=7).

We finally tested whether a phosphomimetic WAVE SD mutant of the VCA domain exhibits a higher protein stability or actin polymerization activity *in vivo*. Here, we used the *Drosophila* wing imaginal disc as an *in vivo* model to measure the effect of the WAVE overexpression. We used the *en*-Gal4 driver, which only induces expression in the posterior compartment of wing imaginal discs, whereas the anterior compartment serves as a negative control (Fig. 7A–E, schematic in F). Expression of an *EGFP* transgene served as an additional negative control (Fig. 7A,A′) and that of *wave* RNAi transgene as a positive control (Fig. 7E,E′) for changes in F-actin levels. We found that phosphomimetic WAVE-SD^5x^ (Fig. 7C,C′) is more stable compared to WAVE-wild type (WAVE-WT (Fig. 7B,B′) and phosphomutant WAVE-SA^5x^ (Fig. 7D,D′,G). Protein levels of WAVE-WT and WAVE-SA^5x^ were not significantly different (Fig. 7G). Moreover, despite the fact that phosphomimetic WAVE-SD^5x^ is more stable than the wild type protein, we found no increased activity (F-actin induction) in WAVE-WT and WAVE SD^5x^ cells (Fig. 7H). Thus, these data suggest that phosphorylation of the VCA domain promotes WAVE stability rather than its actin nucleation activity *in vivo*.

## DISCUSSION

**Fig. 8.**
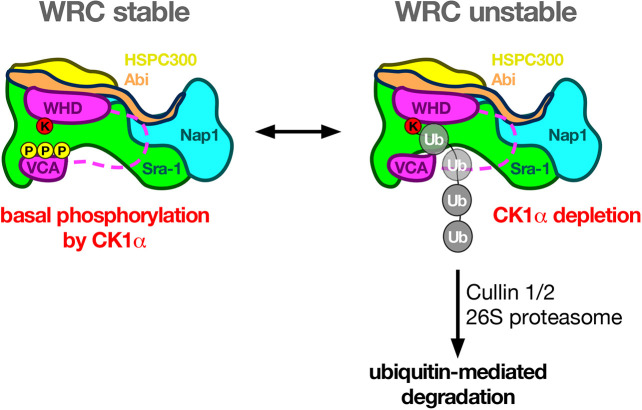
**CK1α protects WAVE from proteasomal degradation.** Schematic showing the proposed role of CK1α in regulating WAVE protein stability. The WRC forms a stable pentameric complex that, in *Drosophila* consists of WAVE (magenta), Abi (orange), Nap1 (blue), Sra-1 (officially known as Cyfip; green), and HSPC300 (yellow). Basal phosphorylation of the VCA domain contributes to protein stability. The VCA domain is normally inhibited through interaction with the meander region of the WAVE WHD domain (Chen et al., 2010) and Sra-1. Release of the autoinhibitory conformation facilitates exposure of WAVE to ubiquitylation, leading to its degradation. Ubiquitylation of WAVE might be mediated by a conserved lysine residue within the WHD domain.

WAVE proteins contain a conserved C-terminal VCA domain that directly binds to and activates the Arp2/3 complex, driving branched actin polymerization. A previous study suggested that CK2 phosphorylates serine of mammalian WAVE2 at positions 482, 484, 488, 489 and 497 within the acidic domain of the VCA domain that promotes Arp2/3 complex activity *in vitro* (Pocha and Cory, 2009). Consistently, overexpression of a GFP-WAVE2 5A construct inhibits lamellipodial protrusion in transfected NIH3T3 cells (Pocha and Cory, 2009). Whether a phosphomimetic WAVE SD mutant of the VCA domain exhibits increased actin polymerization activity *in vitro* has not been addressed so far. In this work, we found that overexpression of phosphomimetic WAVE-SD^5x^ and wild type WAVE equally induces F-actin in epithelial tissue. This suggests that increased stabilization did not necessarily result into increased actin nucleation activity. However, inhibiting the phosphorylation of the VCA domain (see WAVE-SA^5x^ variant) clearly leads to significantly reduced F-actin induction as compared to overexpression of wild type WAVE (WAVE-WT). Therefore, basal phosphorylation of the VCA domain not only seems to be required for protein stability but also seems to promote WAVE activity. However, the phosphomimetic SD^5x^ variant behaves like the wild type WAVE as it fully rescues *wave*-mutant lethality and any defects in lamellipodia formation. Thus, our data more closely resemble previous observations of WAVE2 in NIH3T3 cells (Pocha and Cory, 2009). Our findings also imply that dephosphorylation is a crucial regulatory step in the regulation of WAVE. Our data further suggest that basal phosphorylation by CK1α protects WAVE against increased ubiquitin-dependent degradation. However, the question remains how phosphorylation of the VCA domain by CK1α impacts on WAVE stability? A recent study has demonstrated that WAVE2 undergoes ubiquitylation in a T-cell activation-dependent manner that is followed by proteasomal degradation dependent on the VCA domain (Joseph et al., 2017). The main WAVE2 ubiquitylation site has been mapped to Lys45, a highly conserved residue within the WHD, and is required for WRC integrity and stability (Joseph et al., 2017). A model has been proposed in which activation of WAVE triggers a conformational change that releases the sequestered VCA domain, exposes the WHD (Lys45), to ubiquitylation and, thereby, promotes WAVE degradation (Joseph et al., 2017). Overall, our data provide first *in vivo* evidence for CK1α-dependent phosphorylation of WAVE as a so far unknown and crucial mechanism to control WAVE functions (Fig. 8).

Our data also show that CK1α – but not CK2 – is an important regulator of WAVE function *in vivo*. In contrast to *Ck1α*-mutant cells, macrophages deficient for the catalytic CK2α subunit formed normal lamellipodia but showed increased differentiation of lamellocytes at the expense of macrophages. Our data suggest that CK2 regulates differentiation, rather than shape and locomotion, of blood cells. Accordingly, previous data revealed that phosphorylation of dMi-2, the catalytic subunit of the nucleosome remodeling and deacetylase (NuRD)/Ush complex, by CK2 modulates nucleosome remodeling activity, which might contribute to the repression of blood lineage-specific genes (Bouazoune and Brehm, 2005; Lenz et al., 2021).

## MATERIALS AND METHODS

### *Drosophila* genetics

Fly husbandry and crossing were carried out according to the standard methods. Crosses were maintained at 25°C, UAS-Gal4-based experiments including RNAi were performed at 29°C. The following fly lines were used: CK1α RNAi BL 25786 and VDRC 16645, *hml*Δ-Gal4, UAS-eGFP (Sinenko and Mathey-Prevot, 2004); *en*-Gal4 (Bloomington Stock Center). Transgenic pUASp-*wave* WT, pUASp-*wave* SD^3x^, pUASp-*wave* SA^5x^, pUASp-*wave* SD^5x^ and pUASp-*wave* SA^5x^ flies were generated using ΦC31-mediated transgenesis (*M{3xP3-RFP.attP}ZH-86F* (Bischof et al., 2007).

To generate MARCM-ready stocks the following fly lines from Bloomington Stock Center were used: *hsFLP*, *tubP-GAL80*, w*, *FRT19A*; *Pin*/*CyO*; (BL# 5133); *FRT19A*;; (BL# 1709); *mys^1^FRT19A*/*FM7c*;; (BL# 23862), y^1^ w* Ck1α^[^^A*]*^ FRT19A/FM7c, Kr-GAL4, UAS-GFP (BL#57084), y^1^ w* Ck1α^8B12^ FRT19A/FM7i, twi-GAL4, UASt-GFP; sna^[Sco]^/CyO (BL# 63802); y^1^ w* Ck1α[A] FRT19A/FM7c, Kr-GAL4, UAS-GFP (BL# 64459); hsFLP, w*, y; *tubP-GAL80*, *FRT40A*; *hml*Δ-GAL4, UAS-eGFP/TM6B; *hsFLP; wave^Δ37^, FRT40A/Cyo*; y* w*;; *ck2α*^[TikR]^/TM3 (BL# 24511); y* w*;; *ck2α*^TIK^/TM3 (BL# 24512); y* w*;; *ck2α*^P1^/TM6B, Tb1 (Kyoto 141869). MARCM (Mosaic Analysis with a Repressible Cell Marker; Wu and Luo, 2006) experiments were performed as follows.

Deleted in hemocytes only (DEMON) (Moreira et al., 2013) males were crossed with mutant or FRT19a control virgin flies and placed at 25°C for 48 h. The progeny was submitted to three 1-h heat shocks every 24 h at 37°C. Between heat shocks, crosses were maintained at 29°C. Each heat shock was carried out in a 37°C water bath, followed by 1 h at 18°C to extend the G2 phase and improve MARCM efficiency. Female third-instar larvae containing GFP-expressing hemocytes were then selected for further analysis.

### Cell culture, cell transfection

*Drosophila* S2R^+^ cells were propagated in 1× Schneider's *Drosophila* Medium, as described previously (Stephan et al., 2008). S2R^+^ cells were transfected as described previously (Nagel et al., 2017).

### Cell transfection and maintenance of stable cell line

*Drosophila* S2R^+^ cells were transfected with 11.37 µg pMT-*Ck1α*-6×c-myc plasmid expressing Myc-tagged CK1α (Lam et al., 2018). For selection of co-transfected cells, we used the pCoHygro (Invitrogen) selection vector (1.26 µg) expressing the hygromycin resistance gene. Three days after transfection, medium was replaced and cells were cultured in the presence of 300 μg/ml hygromycin-B (ThermoFisher). Stably transfected cells were screened for hygromycin resistance over 5 weeks. During this time, the medium was changed every 4–5 days. Expression of CK1α was induced by addition of CuSO_4_ (final concentration of 500 µM). Cells were harvested at indicated time points.

### Chemical inhibitors

The CK1α inhibitor D4476 (Sigma) was resuspended in DMSO to 5000 µM stock dilution. D4476 was then diluted in cell culture medium and added to cells at final concentrations of 100 µM in 24-well tissue culture plates. Cells were treated for 4 h. The proteasome inhibitor MG132 (Sigma) was resuspended in DMSO to 1 mM stock dilution. Cells were treated with 10 µM MG132 for 4 h. Control-treated cells were treated with equal volumes of DMSO under conditions identical to those of drug treatment.

### Phosphorylation assays

Phosphorylation assays were conducted using recombinant glutathione S-transferase (GST)-tagged *Drosophila* WAVE protein (GST-WAVE) as substrate (0.2 µg/µl in 50 mM Tris buffer pH 8.0). First, GST-WAVE was incubated with ATPγS. Which serves as the phosphate donor from which mono-thiophosphate instead of phosphate is transferred to the substrate. Second, alkylation of thiophosphorylated serine, threonine or tyrosine residues is allowed by addition of para-nitrobenzylmesylate (PNBM). Alkylated thiophosphate creates an epitope for thiophosphate ester-specific antibodies, which allows to detection phosphorylation (Allen et al., 2007). Kinases used were CK1 (truncated human δ isoform) (Graves and Roach, 1995) and Abl [truncated form of murine leukemia virus v-Abl (Foulkes et al., 1985)], both purchased from NEB. Reactions were mixed and incubated for 30 min at 30°C before PNBM was added for alkylation. Alkylation with PNBM occurred for 120 min at 30°C.

### Co-immunoprecipitation experiments

Co-immunoprecipitation experiments were performed as described previously (Bogdan et al., 2004). Samples were used for SDS PAGE and western blot analysis as described below.

### SDS-PAGE and western blot analysis

Protein extracts were separated by SDS-PAGE and analyzed by western blotting. The following antibodies were used: anti-WAVE (1:1000; Bogdan et al., 2004), anti-Tubulin (1:3000, DSHB AA4.3) and anti-thiophosphate ester (rabbit, ab133473, Abcam). The following secondary antibodies were used diluted 1:5000 in 10% milk TBS-T: goat anti-mouse IgG (H+L), HRP (ThermoFisher) and goat anti-guinea Pig IgG (H+L), HRP. Western blots were quantified using Image studio lite 5.2 software from Li-Cor and statistically analyzed using GraphPad Prism 8 software.

### Immunohistochemistry of *Drosophila* macrophages and wing imaginal discs

Pupal macrophages were isolated as described previously (Ruder et al., 2018). Wing imaginal discs were dissected from third-instar larvae and collected in ice-cold PBS. The discs were fixed with 4% PFA for 45 min at RT on a rotary mixer. 3% (w/v) BSA in PBS, 0.3% Triton X-100 was used as blocking solution. Samples were incubated in primary antibody over night at 4°C. 0.3% PBT was used for every washing step. Primary antibodies used were anti-WAVE (1:1000; Bogdan et al., 2004) and anti-Atilla (1:10; Kurucz et al., 2007). Secondary antibody was goat anti-guinea pig 488 (1:1000, Thermo Fisher Scientific). Actin staining was carried out using Alexa Fluor 568 conjugated to phalloidin (1:100) during the secondary antibody incubation for 2 h. Discs were mounted in Fluoromount G (Thermo Fisher Scientific) and stored at 4°C.

### Image acquisition and microscopy

Structure illumination microscopy images were taken with an ELYRA S.1 microscope (Cell Observer SD, 63×/1.4 oil-immersion objective). Confocal fluorescence images were taken using a Leica TCS SP8 with an HC PL APO CS2 63×/1.4 oil objective. Live imaging of macrophage cultures was performed using a Zeiss CellObserver Z.1 with a Yokogawa CSU-X1 spinning disc scanning unit and an Axiocam MRm CCD camera (6.45 µm×6.45 µm). Ablation experiments were done using a 355 nm pulsed UV laser (Rapp, Optoelectronics), as reported previously (Sander et al., 2013; Rüder et al., 2018).

### Structural protein visualization and analysis

Molecular visualization, editing and analysis, and image production were carried out using the UCSF Chimera package (Pettersen et al., 2021).

### Quantification of *Drosophila* macrophages and analysis of cell morphology

Cell morphology was analyzed by using FIJI shape descriptor parameter. Circularity ranges were between 0 (infinitely elongated polygon) and 100 (perfect circle), 4π×area÷perimeter^2^ (Zdilla et al., 2016).

### Quantification of directed migration of macrophages

Tracking of migrating macrophages was performed using the spots module of Imaris 9.3 (Bitplane; https://imaris.oxinst.com/versions/9-3) software. The reference frame module was set at the ablation site. After automatic tracking, all time-lapse movies were checked and were manually corrected if neccessary. The mean track speed was measured by using the Imaris software and values were analyzed with Graph Pad Prism. The bias angle between the vector towards the ablated cell and the direction vector of the cell was calculated in R software (R Studios Version 1.4). The angle between the vector directly towards the ablation cell and the direction vector of the cell at each time point was calculated using 
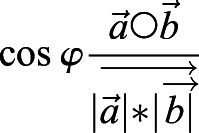
,

i.e. the scalar product 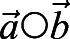 of vectors *a* and *b* divided by the multiplication product of the length of each vector 
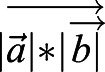
.

Polar histograms were generated using the package ggplot2. For directed migration only cells within a 10–80 µm radius of the wounding site were analyzed. Results were statistically analyzed with GraphPad Prism 8.

### Quantification of actin and WAVE levels in wing imaginal discs

Confocal microscopy images were processed and quantified with FIJI software. F-actin and WAVE intensities were quantified within the same plane. The intensities of three different regions (10×10 µm size) within the posterior and anterior compartment were quantified for each experiment. The integrated density of each square was measured using FIJI software. The mean value of each side was taken to calculate the quotient of posterior over anterior intensity.

### Statistics

Results were statistically analyzed with GraphPad Prism 8.

## Supplementary Material

Supplementary information

Reviewer comments
