# Supplementary material for: CK1α protects WAVE from degradation to regulate cell shape and motility in the immune response
Source: J Cell Sci. 2021 Dec 9;134(23):jcs258891. doi: 10.1242/jcs.258891 (PMC8714073; doi:10.1242/jcs.258891)
Supplement: Supplementary information [file joces-134-258891-s1.pdf]

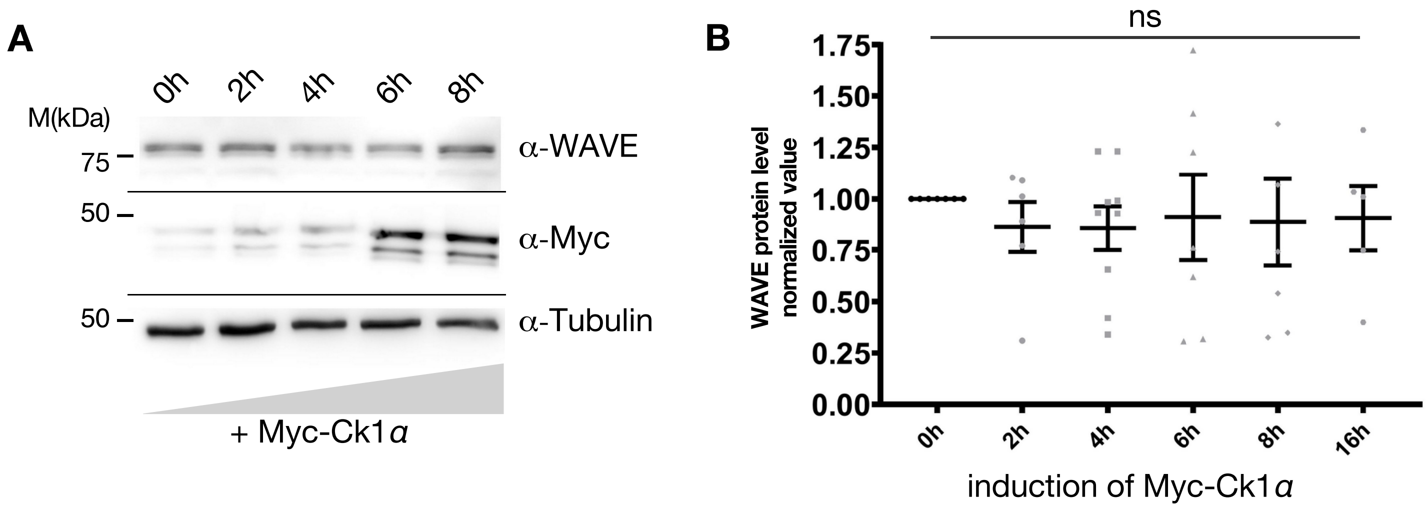

Fig. S1. (A) Induced expression of a myc-tagged CK1α protein in S2 cells. **(B)** Quantification of WAVE protein level upon induced expression. The results are the averages of seven independent experiments. There is no significant increase in WAVE level.

Journal of Cell Science • Supplementary information

Table S1. A list of transgenic RNAi fly lines screened for cell shape changes.

CG number	Gene name	Phenotype	Fly line ID
**CG10023**	Fak56D - Focal Adhesion Kinase	no	VDRC 17957<br>VDRC 108608
**CG10244**	Cadherin96Ca	no	VDRC 1089
**CG10260**	Phosphatidylinositol 4-kinase III alpha	a few spiky cells	BL-35256<br>BL-38242<br>BL-35643<br>VDRC 15993<br>VDRC 105614<br>NIG 10260Rb-1<br>NIG 10260Rb-2
**CG10295**	PAK-kinase	no	VDRC 12553
**CG10564**	Adenylyl cyclase 78C	no	VDRC 51978
**CG10637**	Numb-associated kinase	no	VDRC 35482
**CG10673**	Threonyl-carbamoyl synthesis 5	no	VDRC 35482
**CG10776**	wishful thinking	no	VDRC 865
**CG10895**	loki	no	VDRC 44980
**CG10951**	nimA-like kinase	no	VDRC 16120
**CG10967**	Autophagy-specific gene 1	no	VDRC 16133
**CG1107**	auxillin	no	VDRC 103426<br>VDRC 16182
**CG11228**	hippo	no	VDRC 7823
**CG11420**	pan gu	no	VDRC 31500
**CG11489**	serine-arginine protein kinase at 79D	no	VDRC 47544
**CG11533**	Asator	no	VDRC 47544
**CG11660**	CG11660	no	VDRC 18526
**CG12066**	cAMP-dependent protein kinase 2	no	VDRC 30685
**CG12069**	CG12069	no	VDRC 23719
**CG1210**	Phosphoinositide-dependent kinase 1	no	VDRC 18736
**CG12147**	CG12147	no	VDRC 31658<br>VDRC 31659
**CG1227**	CG1227	no	VDRC 38647
**CG12306**	polo	no	VDRC 20177
**CG13388**	A kinase anchor protein 200	no	VDRC 5647<br>VDRC 102374
**CG14026**	thickvein	no	VDRC 105834

Journal of Cell Science • Supplementary information

**CG14030**	bub1 homologue	spiky cells	BL-35260<br>VDRC 101096<br>VDRC 24833<br>NIG 14030R-1<br>NIG 14030R-2
**CG14080**	Mitogen-activated protein kinase phosphatase 3	no	VDRC 45415
**CG14217**	Tao	no	VDRC 17432
**CG14226**	domeless	no	VDRC 19717
**CG14305**	CG14305	no	VDRC 17477
**CG14895**	Pak3	no	VDRC 107260
**CG14992**	Ack	no	VDRC 39857
**CG1506**	Ac3	no	VDRC 33217
**CG15224**	Casein kinase 2 β subunit	a few spiky cells, more lamellocytes	BL-31254<br>VDRC 32377<br>VDRC 106845<br>VDRC 32378
**CG15793**	Downstream of raf1	no	VDRC 40025
**CG15862**	cAMP-dependent protein kinase R2	no	VDRC 101763<br>VDRC 39436<br>VDRC 39437
**CG1594**	hopscotch	no	VDRC 40037
**CG17090**	homeodomain interacting protein kinase	no	VDRC 32854
**CG17161**	grapes	no	VDRC 11076
**CG17174**	ACXB	no	VDRC 9748
**CG17216**	KP78b	no	VDRC 51996
**CG17256**	Nek2	no	VDRC 40052
**CG17348**	derailed	no	VDRC 3047
**CG17520**	Casein kinase 2 α subunit	more lamellocytes	BL-31645<br>BL-35136
**CG17596**	Ribosomal protein S6 kinase II	no	VDRC 5702<br>VDRC 101451
**CG17998**	G protein-coupled receptor kinase 2	no	VDRC 1835
**CG18069**	Calcium/calmodulin-dependent protein kinase II	no	VDRC 47280
**CG18247**	SH2 ankyrin repeat kinase	no	VDRC 105706
**CG1830**	Phosphorylase kinase γ	no	VDRC 33054
**CG18402**	Insulin-like receptor	no	VDRC 991
**CG1848**	LIM-kinase1	no	VDRC 25344

Journal of Cell Science • Supplementary information

			VDRC 25343
**CG18582**	mushroom bodies tiny	no	VDRC 46043
**CG1951**	CG1951	no	VDRC 33431
**CG1954**	Protein C kinase 98E	no	VDRC 33434
**CG1973**	yata	no	VDRC 19275
**CG2028**	Casein kinase 1α	many spiky cells, stellate	BL-35152<br>BL-35153<br>BL-41711<br>BL-25786<br>VDRC 110768<br>VDRC 13664
**CG2048**	discs overgrown	no	VDRC 9241
**CG2049**	Protein kinase related to protein kinase N	no	NIG 2055R-1
**CG2079**	Downstream of kinase	no	VDRC 20796<br>VDRC 108544<br>VDRC 20796
**CG2252**	female sterile (1) homeotic	no	VDRC 51227
**CG2272**	slipper	no	VDRC 33516
**CG2577**	CG2577	no	VDRC 41693
**CG2615**	IκB kinase-like 2	no	VDRC 12485
**CG2845**	pole hole	no	VDRC 20909<br>VDRC 107766
**CG2899**	kinase suppressor of ras	no	VDRC 45040
**CG3008**	CG3008	no	VDRC 52634
**CG3051**	SNF1A/AMP-activated protein kinase	no	VDRC 1827<br>VDRC 106200
**CG3086**	MAP kinase activated protein-kinase-2	no	VDRC 3170
**CG3105**	PAS kinase	no	VDRC 25661
**CG31421**	Tak1-like1	no	VDRC 25760
**CG32019**	bent	no	VDRC 46253
**CG32031**	Arginine kinase	no	VDRC 34037
**CG32134**	breathless	no	VDRC 27106
**CG3216**	CG3216	no	VDRC 29915

Journal of Cell Science • Supplementary information

**CG32417**	Myt1	no	VDRC 34547
**CG3249**	spoonbill	no	VDRC 48005<br>VDRC 48006
**CG3277**	CG3277	no	VDRC 7271
**CG3319**	Cyclin-dependent kinase 7	no	VDRC 10442
**CG34361**	Diacyl glycerol kinase	no	VDRC 38239
**CG34412**	Tousled-like kinase	a few spiky cells, some stellate	BL-33983<br>BL-35298<br>BL-36102
**CG3682**	PIP5K59B	no	VDRC 47027
**CG3915**	Derailed 2	no	VDRC 40484
**CG4007**	Neurospecific receptor kinase	no	VDRC 36282
**CG4012**	genghis khan	no	VDRC 107207<br>VDRC 28367
**CG4032**	ABL- tyrosine kinase	no	VDRC 2897
**CG4041**	CG4041	no	VDRC 34780
**CG4141**	Pi3k92E	no	BL-27690<br>VDRC 38986<br>VDRC 107390<br>VDRC 38986<br>VDRC 107390<br>VDRC 38985
**CG4154**	Guanylyl cyclase at 88E	no	VDRC 21797
**CG4201**	immune response deficient 5	no	VDRC 26427
**CG42341**	cAMP-dependent protein kinase R1	no	VDRC 103303<br>VDRC 26329<br>VDRC 26328<br>VDRC 103720<br>VDRC 103303<br>VDRC 26329<br>VDRC 26328<br>VDRC 103720
**CG42349**	Protein kinase C δ	spiky cells cells smaller	VDRC 101029<br>VDRC 31468<br>VDRC 33837<br>VDRC 101421<br>VDRC 31468<br>VDRC 22755<br>VDRC 33838
**CG4252**	meiotic 41	no	VDRC 11251

**CG4268**	Pitslre	no	BL-35157<br>BL-56855<br>VDRC 107303
**CG43143**	Nuak family kinase	no	VDRC 16334
**CG43217**	C-terminal Src kinase	no	VDRC 32877<br>VDRC 102313<br>VDRC 48282<br>VDRC 48281
**CG4379**	cAMP-dependent protein kinase 1	no	VDRC 101524
**CG44012**	Btk family kinase at 29A	no	VDRC 106962
**CG4488**	wee	no	VDRC 26543
**CG4551**	Dyrk2 - Dual-specificity tyrosine phosphorylation-regulated kinase 2	no	VDRC 40534
**CG4583**	Ire1 - Inositol-requiring enzyme-1	no	VDRC 39561
**CG4803**	Takl2 - Tak1-like 2	no	VDRC 34898
**CG4839**		no	VDRC 26641
**CG4926**	Ror - Ror	no	VDRC 935
**CG5072**	Cdk4 - Cyclin-dependent kinase 4	no	VDRC 40577
**CG5179**	Cdk9 - Cyclin-dependent kinase 9	no	VDRC 30448
**CG5182**	Pk34A - Pk34A	no	VDRC 27368
**CG5206**	bon - bonus	no	VDRC 44284
**CG5363**	cdc2 - cdc2	a few spiky cells	VDRC 106130<br>VDRC 41838
**CG5408**	trBL- - tribBL-es	no	VDRC 22114
**CG5483**	Lrrk - Leucine-rich repeat kinase	no	VDRC 22139
**CG5680**	bsk - basket	no	VDRC 104569<br>VDRC 34139
**CG5790**		no	VDRC 45045
**CG5974**	pll - pelle	no	VDRC 2889
**CG5983**	ACXB - ACXB	no	VDRC 2870
**CG6027**	cdi - center divider	no	VDRC 43634
**CG6033**	drk - downstream of receptor kinase	no	VDRC 105498

Journal of Cell Science • Supplementary information

**CG6114**	sff - sugar-free frosting	no	VDRC 22225
**CG6355**	fab1	no	VDRC 27591
**CG6498**		no	VDRC 109282<br>VDRC 35101
**CG6518**	inaC - inactivation no afterpotential C	no	VDRC 2895<br>VDRC 2894
**CG6535**	tefu - telomere fusion	no	VDRC 22502
**CG6551**	fu - fused	no	VDRC 27663
**CG6620**	ial - Ipll-aurora-like kinase	no	VDRC 35107
**CG6622**	Pkc53E - Protein C kinase 53E	no	VDRC 27696<br>VDRC 27699
**CG6703**	CASK - CASK ortholog	no	VDRC 34184
**CG6715**	KP78a - KP78a	no	VDRC 51616<br>VDRC 26722<br>VDRC 47658<br>VDRC 47657
**CG6963**	gish - gilgamesh	spiky cells<br>cells smaller	VDRC 26003<br>VDRC 106826<br>VDRC 26003<br>VDRC 106826
**CG7004**	four wheel drive	no	VDRC 27786<br>VDRC 27785
**CG7097**	happyhour	no	VDRC 35166
**CG7111**	Receptor of activated protein kinase C 1	no	VDRC 27859<br>VDRC 27858
**CG7156**	CG7156	no	VDRC 26035
**CG7177**	Wnk	no	VDRC 35193
**CG7180**	CG7180	no	VDRC 34369
**CG7186**	Sak kinase	no	VDRC 27904
**CG7223**	heartless	no	VDRC 27180<br>VDRC 6692<br>VDRC 27180
**CG7524**	Src oncogene at 64B	no	VDRC 35252
**CG7597**	cdk 12 – Cyclin-dependent kinase 12	spiky cells,<br>cells smaller	BL-35163<br>BL-34838<br>BL-42775<br>VDRC 25508

Journal of Cell Science • Supplementary information

			VDRC 25510
**CG7693**	frayed	no	VDRC 41718
**CG7717**	Mekk1	no	VDRC 25529
**CG7838**	Bub1-related kinase	no	VDRC 26109
**CG7873**	Src oncogene act 42A	no	VDRC 26019
**CG7892**	nemo	no	VDRC 3002
**CG8174**	SRPK	no	VDRC 26933
**CG8201**	par-1	no	VDRC 52553<br>VDRC 52556
**CG8203**	Cyclin-dependent kinase 5	no	VDRC 35856<br>VDRC 35855<br>VDRC 104491
**CG8222**	PDGF- and VEGF-receptor related	no	VDRC 976<br>VDRC 43461<br>VDRC 13502<br>VDRC 43459<br>VDRC 105353<br>VDRC 977
**CG8250**	Alk	no	VDRC 11446
**CG8485**	CG8485	no	VDRC 35940
**CG8726**	CG8726	no	VDRC 40719
**CG8767**	mos	no	VDRC 36531
**CG8789**	wallenda	no	VDRC 26910
**CG8808**	Pyruvate dehydrogenase kinase	no	VDRC 37966
**CG8874**	Fps oncogene analog 85D	no	VDRC 107266<br>VDRC 36053<br>VDRC 36054
**CG8878**	CG8878	no	VDRC 28970
**CG8967**	off-track	no	VDRC 30833
**CG9210**	Adenylyl cyclase 35C	no	VDRC 11547
**CG9222**	CG9222	no	VDRC 27010
**CG9533**	rutabaga	no	VDRC 101759<br>VDRC 5569
**CG9738**	MAP kinase kinase 4	no	VDRC 26929

Journal of Cell Science • Supplementary information

**CG9774**	Rho-kinase	cytokinesis defects	BL-35305<br>BL-34324<br>BL-28797<br>VDRC 3793<br>VDRC 104675<br>NIG 9774R-2<br>NIG 9774R-3
**CG9962**	CG9962	no	VDRC 36473<br>VDRC 36178<br>VDRC 108721
**CG9985**	skittles	no	BL-35198<br>BL-27715<br>BL-101624<br>VDRC 6229

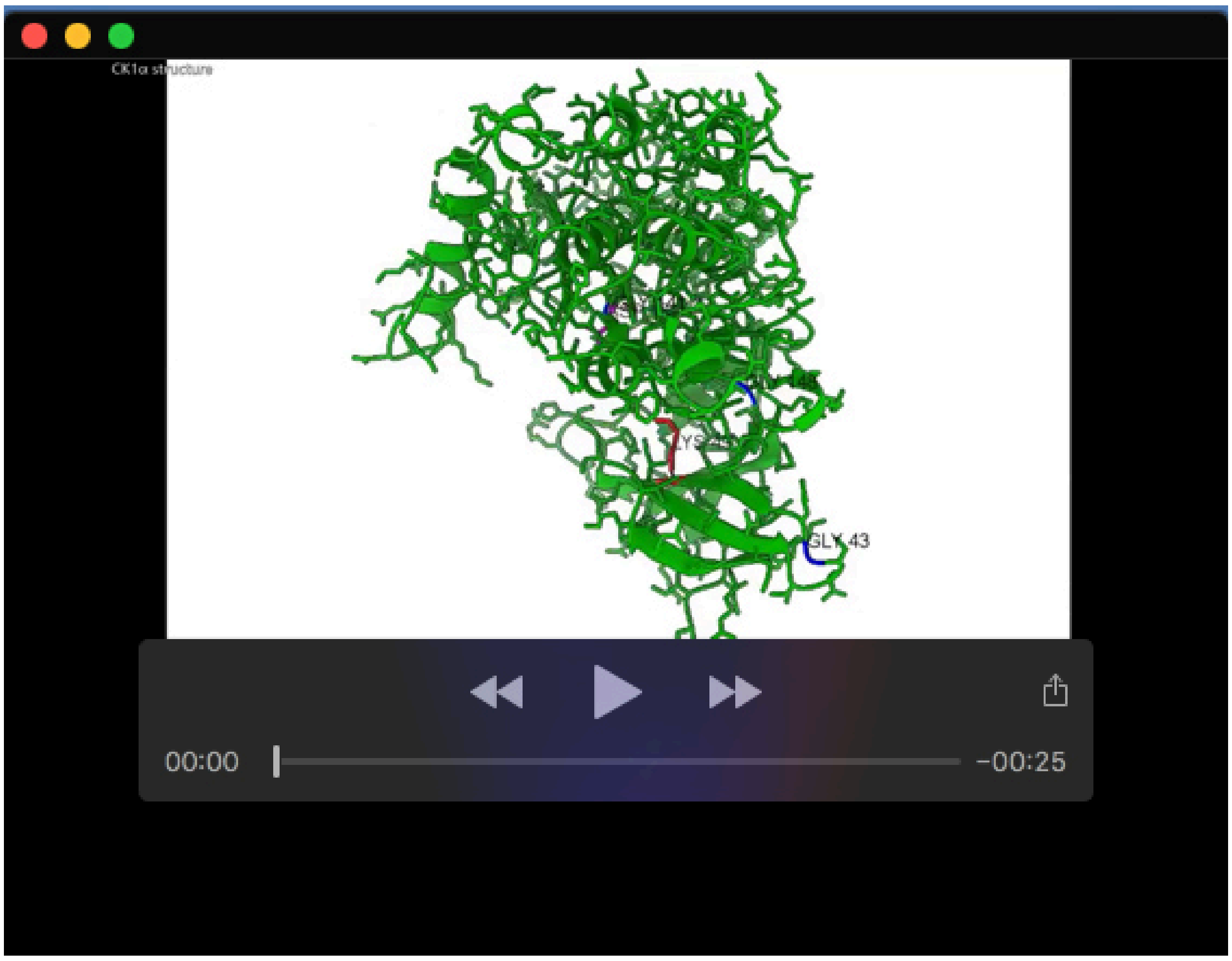

Movie 1. Representation of the three-dimensional (3D) structure of CK1α using the UCSF Chimera software (Pettersen et al., 2021). The ATP-binding Site (Lysin49) is highlighted in red; the active site/ proton acceptor (Asp139) is highlighted in magenta. Residue glycine 43 ($ck1\alpha^{G43D}$), glycine 148 ($ck1\alpha^{G148S}$) and lysine 141 ($ck1\alpha^{L141M}$) that are replaced in the three different available mutant *ck1α* alleles are highlighted in blue. Subsequently, the mutated structure is depicted. The substitution lysine to methionine at position 141 removes an H-bond and favors unfavorable interactions with the active site where atoms are too close together (dashed lines in magenta), highlighted in the last sequence of the video. Labels are located on the upper left side.

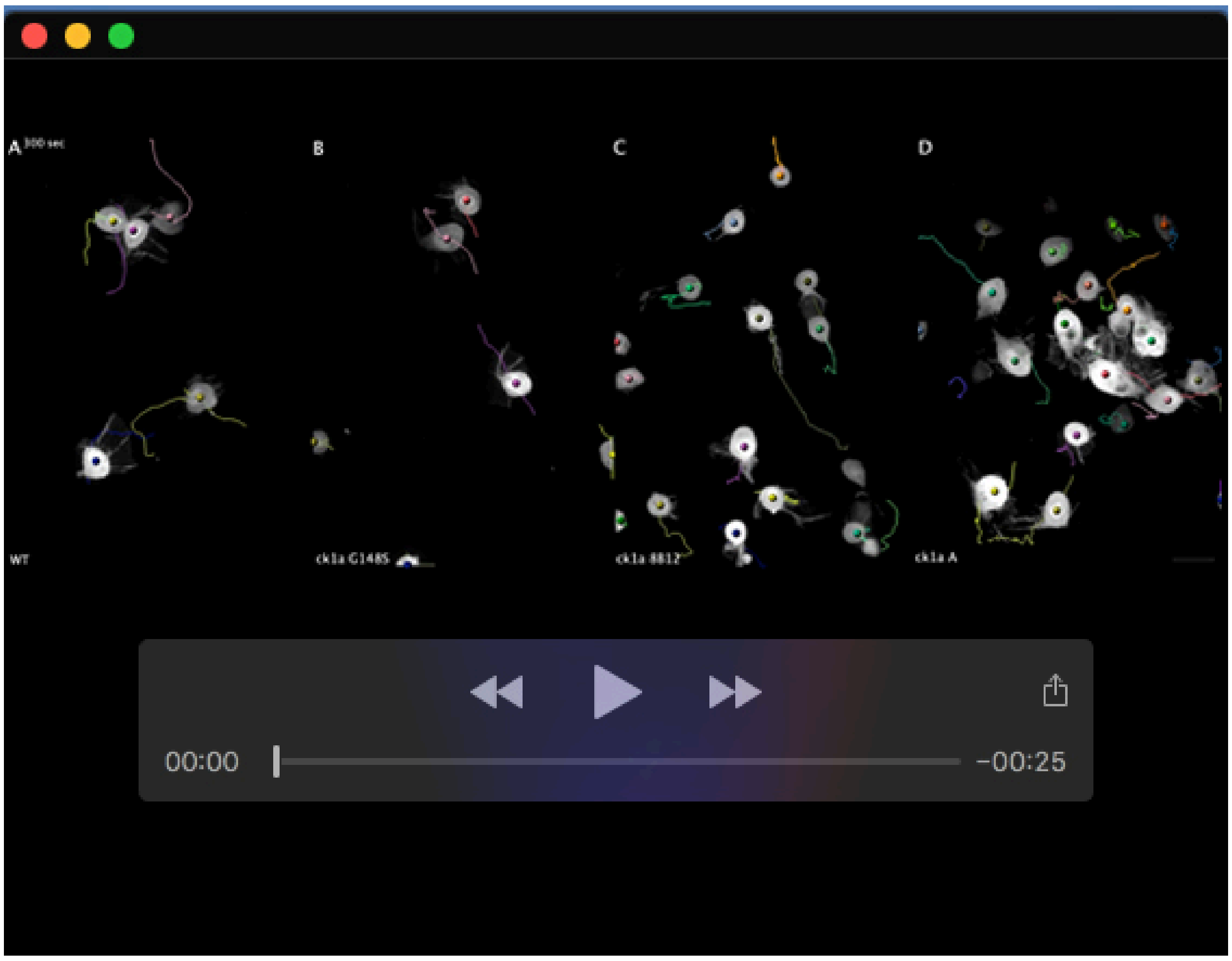

Movie 2. Spinning disc microscopy videos of randomly migrating pupal **(A)** WT wild type, **(B)** *ck1α* ^{G148S} mutant, **(C)** *ck1α* ^{G43D} mutant and **(D)** *ck1α* ^{L141M} mutant macrophages expressing an EGFP transgene imaged from a living prepupa (2 h APF). Migratory tracks of individual cells are indicated (colored, jagged lines). Scale bars represent 10 µm.

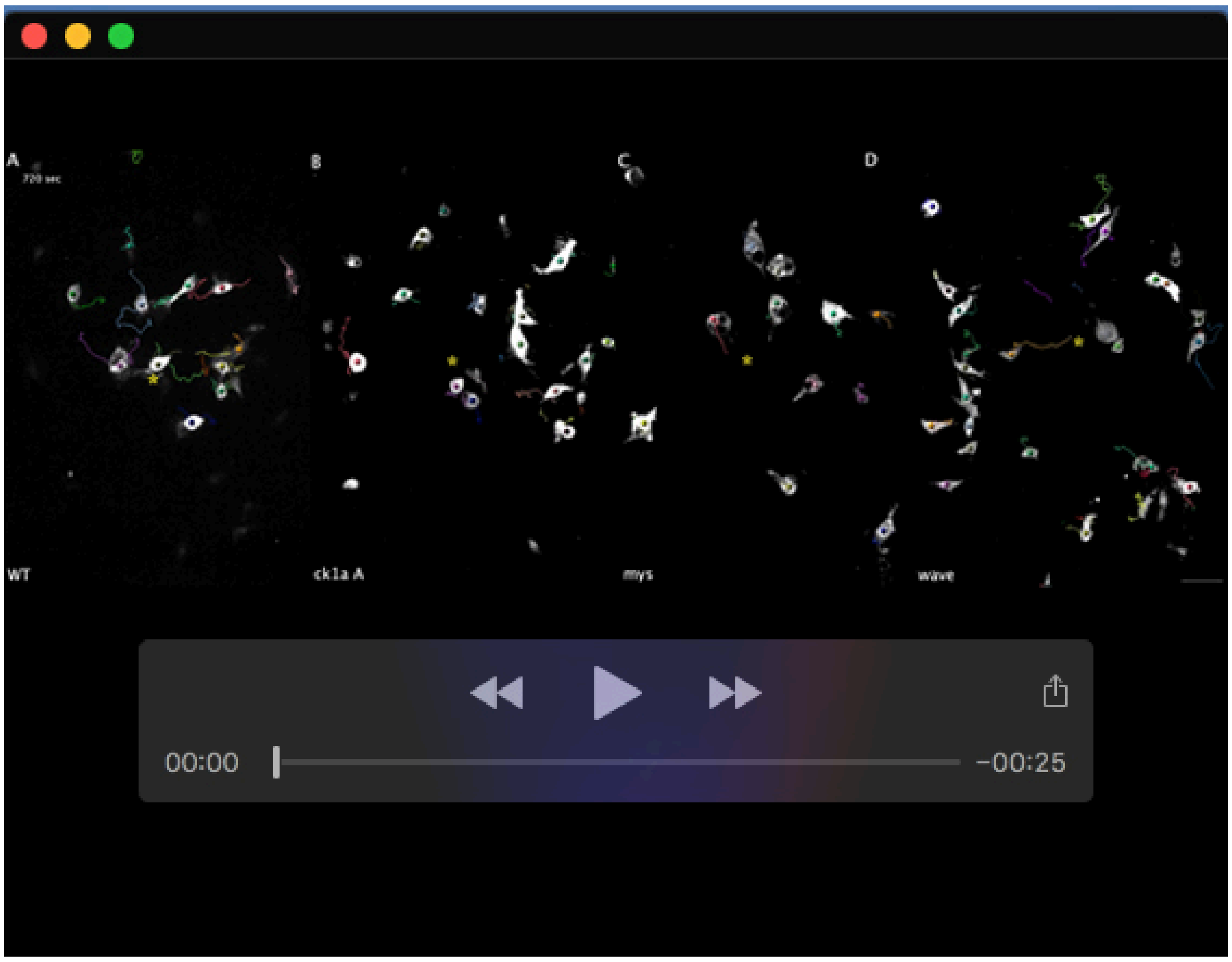

Movie 3. (A-D) Spinning disc microscopy videos of macrophages that migrate towards a laser-ablated cell (indicated by the yellow circle). Cells are imaged for 30 minutes after ablation in a 30 seconds interval and tracked afterwards using Imaris. (**A**) WT wild type (**B**) $ck1\alpha^{L141M}$ mutant, (**C**) $wave^{\Delta 37}$ and (D) βPS-integrin (mys^1) mutant macrophages. Homozygous mutant cells are labeled by GFP expression using the MARCM system. Scale bars represent 10 μm.
