# Supplementary material for: CK1α protects WAVE from degradation to regulate cell shape and motility in the immune response
Source: J Cell Sci. 2021 Dec 9;134(23):jcs258891. doi: 10.1242/jcs.258891 (PMC8714073; doi:10.1242/jcs.258891)
Supplement: Reviewer comments [file joces-134-258891_review_history.pdf]

CK1α protects WAVE from degradation to regulate cell shape and motility in immune response

Alexander Hirschhäeuser, Marianne van Cann and Sven Bogdan
DOI: 10.1242/jcs.258891

Editor: Michael Way

Review timeline
Original submission: 10 May 2021
Editorial decision: 14 June 2021
First revision received: 29 September 2021
Editorial decision: 19 October 2021
Second revision received: 23 October 2021
Accepted: 25 October 2021

Original submission

First decision letter

MS ID#: JOCES/2021/258891

MS TITLE: A novel role of CK1Î± in regulating cell shape and motility through WAVE in vivo

AUTHORS: Alexander Hirschhaeuser, Marianne van Cann, and Sven Bogdan
ARTICLE TYPE: Research Article

We have now reached a decision on the above manuscript.

To see the reviewers' reports and a copy of this decision letter, please go to: https://submit-jcs.biologists.organd click on the 'Manuscripts with Decisions' queue in the Author Area. (Corresponding author only has access to reviews.)

As you will see, the reviewers are positive but raise a number of substantial criticisms that prevent me from accepting the paper at this stage. They suggest, however, that a revised version might prove acceptable, if you can address their concerns. In particular, I think you need to provide more details and clarity of what has actually been done as the reviewers found it hard to follow the experiments and the analysis in several places. In addition, I think it is also essential that you provide somein vivo demonstration in flies of phosphorylation-mediated WAVE stability given your title. If you think that you can deal satisfactorily with the criticisms on revision, I would be pleased to see a revised manuscript. We would then return it to the reviewers.

We are aware that you may be experiencing disruption to the normal running of your lab that makes experimental revisions challenging. If it would be helpful, we encourage you to contact us to discuss your revision in greater detail. Please send us a point-by-point response indicating where you are able to address concerns raised (either experimentally or by changes to the text) and where you will not be able to do so within the normal timeframe of a revision. We will then provide further guidance. Please also note that we are happy to extend revision timeframes as necessary.

Please ensure that you clearly highlight all changes made in the revised manuscript. Please avoid using 'Tracked changes' in Word files as these are lost in PDF conversion.

© 2021. Published by The Company of Biologists under the terms of the Creative Commons Attribution License (https://creativecommons.org/licenses/by/4.0/).

I should be grateful if you would also provide a point-by-point response detailing how you have dealt with the points raised by the reviewers in the 'Response to Reviewers' box. Please attend to all of the reviewers' comments. If you do not agree with any of their criticisms or suggestions please explain clearly why this is so.

Reviewer 1

Advance summary and potential significance to field

The authors dissect a role for CK1alpha in controlling WAVE function in Drosophila macropahges. They reveal that WAVE is a likely target of CK phosphorylation and suggest that this phosphorylation event is important for WAVE activity. As little appears to be known about WAVE phosphorylation the work will be of interest to the community.

Comments for the author

Some of the data in the manuscript are unclear and need improving. Additionally, they suggest that the mechanism of CK1 action on WAVE is to protect it from degradation. However, this is not really based on much data.
Comments:
- Please add line numbers to make it easier for reviewers to comment
- In the text the authors never state whether the RNAi screen was done in vitro or in vivo? Additiionally, is Figure 1 in vitro??
- Any comment on why the G148S mutant does now show a migration phenotype but does show a difference in cell shape? Is this a weak mutant?
- In Figure 2E they measure a bias angle?? What is this metric?? Its not an obvious migration parameter.
Why not quantify numbers of hemocytes at the wound or something easier to interpret?
- It is unclear what 2F is measuring. The angles don't look any different in the rose plot. Is there some metric that you could measure that can be statistically tested?
- Can 2I and 2J be quantified? One of their possible conclusions is that WAVE phosphorylation prevents its degradation and this result should be measured.
- The lethality experiments in 3B should be statistically compared.
- In the text "Siimilarly, we still found significant defects in the migratory behaviour of mutant macrophages…..(quantification in figure 3D)"…. Is this a typo? 3D is measuring circularity, not migration??
- Can 4A be quantified?
- In the discussion the authors introduce additional data (4C and 4D). Please move to results.
- In the discussion they state that basal phosphorylation by CK protects WAVE against ubiquitin dependent degradation. Is there a reason they did not look at WAVE amount in hemocytes in vivo (or isolated from larvae) in the background of a CK mutant or CK RNAi (or even in whole animals)? This conclusion begs this experiment.

Reviewer 2

Advance summary and potential significance to field

The Arp2/3 complex is one of the cell's major actin nucleators, shaping the cytoskeleton in many ways including driving formation of lamellipodia in migrating cells. The WAVE complex is a critical regulator of Arp2/3. Here the authors provide new insights into the regulation of WAVE by phosphorylation, with a focus on casein kinase 1 alpha. They make superb use of the Drosophila hemocyte model, allowing them to combine RNAi and classical mutants and providing assays in vivo. Building on earlier work in vitro and in Dictyostelium, they find a striking effect of loss of casein kinase 1 alpha on both actin-based protrusions and motility. They identify potential CK1 phosphorylation sites in WAVE and via mutational analysis provide strong support for the idea that basal phosphorylation of sites in the VCA domain are important for WAVE stability and thus function. The data are generally lovely, well quantified and the conclusions well supported. I think this will be of broad interest to cell and developmental biologists interested in cytoskeletal

© 2021. Published by The Company of Biologists under the terms of the Creative Commons Attribution License (https://creativecommons.org/licenses/by/4.0/).

regulation. I did think there were places where the story was presented too rapidly and thus was hard to follow, and also think a few conclusions need to be further quantified or toned down. These issues are outlined below and should be straightforward to address.

Comments for the author

1. The manuscript starts with mention of a genetic screen of candidate genes using RNAi, and include a supplementary table mentioning the many candidates. This was described in a very cursory fashion. If this is because the screen itself will be published elsewhere, the authors should state that directly.
Otherwise they should tell us more—for example was ck1alpha the only hit? Also relevant—was CK2 included in the screen?

2. There were several places where data was referred to I could not find. The first was p. 5, line 12-14.
Where are the data about “impaired spread morphology—if it is supposed to be apparent in the images they need to point this out, perhaps indicating particular cells. The also should explicitly state that the effect on migration was only seen with 2 of the 3 alleles tested—this is OK as they have different lesions but it needs to be stated.

3. The directional migration assay is an excellent one, but it is poorly described/displayed. Is the image of cells shown the initial image? Why do only a subset of the tracks start/end with a cell? Please indicate the start/end=direction of each trajectory, especially those that do not include an imaged cell. They also state “A similar, but more severely impaired migratory behavior showed wave and ßPS-integrin mutant macrophages (Figure 2C, D; Moreira et al., 2013.” but the quantification in panel 2E does not show a difference in severity. Finally, are the wave and ßPS-integrin mutant macrophages from MARCM?

4. The phosphorylation assay used in Fig 2H should be briefly described in the results for those like me not familiar with this assay.

5. The “slight increase” in WAVE levels after CK1alpha overexpression was not obvious—this statement should either be quantified or removed.

6. A reference should be given and a few more details provided documenting the specificity of the CK1alpha inhibitor D4476.

7. A little more clarity in the description of the “consensus motif” S/T-X-X-(X)-S/T would help—it isn’t clear to me which of the S/T residues in the cluster they suggest match this motif. A minor point but it could be clearer. I get that it might have “moved” between flies and mammals.

8. On page 7 they state: “Cells expressing the SA5x variant still showed a strongly reduced circularity compared to cells rescued either by wild type, SA3x or SD5x protein (quantification in figure 3D). Similarly we still found significant defects in the migratory behavior of mutant macrophages expressing phosphomutant SA5x protein compared to wild type or phosphomimetic SD5x variant (quantification in figure 3D).” The former quantification is presented but the latter seems to be missing. In this same part of the manuscript, they should note and comment on the fact that the wildtype WAVE did not rescue the effect of ck1 RNAi (Fig. 3E)—this makes sense once you think about it but needs to be laid out more clearly.

9. Are the actin images in Figure 4A scaled differently? It was odd that there was “more” actin in the control anterior compartment in one than the other.

10. The thing I found most confusing was the data in the rest of Figure 4, which is presented, for unexplained reasons, in a very rambling way and in the Discussion. This should probably be in the Results likely as Figure 2, should be explained much better (only a handful of fly immunologists would understand it) and needs to be coupled with mentioning whether CK2 came out of the original screen. The fact that they are very different enzymes (bottom of p 8) would be the preface for this.

© 2021. Published by The Company of Biologists under the terms of the Creative Commons Attribution License (https://creativecommons.org/licenses/by/4.0/).

Minor issues
p. 5, line 13. Typo—"Figure 1H,I,J"

Reviewer 3

Advance summary and potential significance to field

Hirschhauser et al. investigated how the casein kinase 1α (CK1α) regulates Wave to control cell shape and migration. Using Drosophila macrophages as a model system, the authors first showed that genetic perturbation of ck1α impairs lamellipodial formation and migratory speed in primary culture condition and prepupal wound response in vivo. Subsequent biochemical analysis demonstrated that CK1α can interact with and phosphorylate WAVE, which protects WAVE from ubiquitin-mediated degradation. With further genetic analysis of putative phosphorylation sites in the N- and C-terminal regions of WAVE, the authors identified a cluster of C-terminal acidic residues critical for WAVE function in actin polymerization, lamellipodial formation, cell motility as well as embryonic development.
Overall, the authors made an important discovery of CK1α-mediated WAVE phosphorylation, which protects WAVE from degradation. Experiments are well designed and the data largely support the authors' conclusions. A major concern is the lack of in vivo evidence that supports phosphorylation-mediated WAVE stability.

Comments for the author

Specific comments:
1. Lack of in vivo demonstration of phosphorylation-mediated WAVE stability.
One of the most exciting and novel findings of the manuscript is CK1α-mediated WAVE phosphorylation, resulting in the protection of WAVE from ubiquitin-mediated degradation. However, this is only shown in a biochemical assay using S2 cells. It is unclear if this also occurs in vivo. In particular, the expression level of WAVE-SA5X appears to be similar to that of WAVE-SD5X instead of lower (Fig 4A). Quantification is necessary here. The authors may consider performing genetic interaction studies between proteasome components and WAVE rescue embryos and/or protein stability assays in embryos by western blots and/or immunofluorescence staining.

2. Incomplete investigation and documentation of cell motility phenotype.
The authors used circularity as a readout of lamellipodial formation for most genetic perturbation conditions. Track speed mean for cell motility should also be done for most genetic perturbation conditions. Currently, this assay is not done for the ck1α RNAi and WAVE phospho-mutant studies.

Minor concerns:
1. Fig 1 and text: for all three ck1α alleles used in the study, it would be more consistent to use molecular lesion to mark the alleles (e.g. change [8B12] to [G43D], [A] to [L141M] in figures and texts).
2. ck1α[8B12] may not necessarily be an amorph, but instead a strong hypomorph according to the previous study. Also, ck1α[A] is stronger than ck1α[8B12] in Fig 1. Does this mean that ck1α[A] could be an antimorphic allele?
3. Fig 1 does not show images for each genetic conditions used in the quantification. Please include images for ck1α RNAi #1, wave RNAi, ck1α[8B12]
and ck1α[G148S].
4. In Fig 2G-I, quantify all blots. Show all data points.
5. In Fig 2K, show all data points.
6. In Fig 3B, why WAVE SD3X is more viable than WT?
7. In Fig 3C and text, please clarify that these experiments are performed in wave[Δ3&
7] mutant background and include SD3X image. Fig 3C is not referred in the text.
8. In Fig 3D, include SD3X data.
9. It would be nice to have a model figure to illustrate the conclusion.

© 2021. Published by The Company of Biologists under the terms of the Creative Commons Attribution License (https://creativecommons.org/licenses/by/4.0/).

First revision

Author response to reviewers' comments

Dear Michael,
Thank you very much for considering a revised version of our manuscript entitled "**CK1α protects WAVE from degradation to regulate cell shape and motility in immune response**" (Ms. No. JOCES/2021/258891) and the reviewers for their very positive and very constructive comments.

Let me briefly highlight the **most important points** that we have addressed before I provide a detailed point-to-point- response to the referees' comments.

We have added substantial new data to our story and correspondingly modified the main text. The new experimental data sets include new genetic studies and numerous new quantifications, we thus included 3 new main figures and 1 new supplementary figure. As you will see, we addressed all concerns of the referees. Most importantly, we provided further *in vivo* evidence for a role of CK1α in protecting WAVE from ubiquitin-mediated proteasomal degradation, which was a main criticism of our work by the referees. In detail, we now show:

1. RNAi-mediated CK1*a* depletion resulted in substantial reduction of endogenous WAVE protein level in macrophages .

2. We have screened for proteasome components and tested various RNAi lines including members of the cullin protein family as part of E3 ligase complexes, but also the 26S proteasome. We tested all six known Cullins (Cul1-6) as well as four components of the 20S catalytic core (Prosβ5, Prosβ7) and a 19S regulatory complex (Rpn1, Rpn6). RNAi mediated knockdown of Cul1 and 2, but not Cul3-6 rescued cell shape defects of macrophages evoked by *ck1a* RNAi. Knockdown of any Cullin alone did not show a significant difference in cell shape. Likewise, when we affected the final step of proteasomal degradation by targeting either 20S catalytic core (Prosβ5, Prosβ7) or a 19S regulatory complex (Rpn11 and Rpn6) we found a significant rescue of the spiky macrophage phenotype evoked by *ck1a* RNAi.

3. We carefully compared and quantified in more detail the overexpression of wild type and phosphomutant WAVE variants in wing disc as an *in vivo* model. Our data indicates that the overexpression of both wild type and phospho-mimetic WAVE SD^{5x} but not the phospho-mutant WAVE SA^{5x} of the VCA domain substantially induces F-actin. We could also confirm that WAVE SD5x is more stable compared to WAVE WT and WAVE SA5x.

Below I provide a detailed response to the referees' comments.

Best wishes
Sven

Reviewer 1 Advance Summary and Potential Significance to Field:

The authors dissect a role for CK1alpha in controlling WAVE function in Drosophila macrophages. They reveal that WAVE is a likely target of CK phosphorylation and suggest that this phosphorylation event is important for WAVE activity. As little appears to be known about WAVE phosphorylation the work will be of interest to the community.

We agree and thank the reviewer for pointing out this.

Reviewer 1 Comments for the Author: Some of the data in the manuscript are unclear and need improving. Additionally, they suggest that the mechanism of CK1 action on WAVE is to protect it from degradation. However, this is not really based on much data.

We agree and now provided new *in vivo* evidence for a role of CK1alpha in protecting WAVE from degradation (see below).

© 2021. Published by The Company of Biologists under the terms of the Creative Commons Attribution License (https://creativecommons.org/licenses/by/4.0/).

Comments:
-Please add line numbers to make it easier for reviewers to comment

We now included line numbers in the manuscript.

In the text the authors never state whether the RNAi screen was done in vitro or in vivo? Additiionally, is Figure 1 in vitro??

The reviewer is correct and we apologize for our unclear description. The RNAi screen was done *in vivo* using transgenic RNAi flies. The initial phenotypic screen was done ex vivo with macrophages isolated from larvae, plated on cover slips and stained with phalloidin and DAPI. We now included the following information (see line 131-132: "Macrophages were isolated from third instar larvae and tested for their phenotypic effects on lamellipodia formation and cell spreading *ex vivo*."

-Any comment on why the G148S mutant does now show a migration phenotype but does show a difference in cell shape? Is this a weak mutant?

Yes indeed, this would be our conclusion. The three different *ck1a* alleles bear distinct missense mutations, at glycine 43 ($ck1a^{8B12}$), glycine 148 ($ck1a^{G148S}$, also known as $ck1a^{B}$) and lysine 141 ($ck1a^{A}$). The substitution of the lysine to methionine at position 141 ($ck1a^{A}$) is the only mutation that removes an H-bond affecting the active site of the CK1*a* (see three-dimensional structure in supplementary movie M1, dashed lines). By contrast, replacement of either glycine 43 ($ck1a^{8B12}$) or glycine 148 ($ck1a^{B}$) do not led to any obvious structural changes.
$ck1a^{8B12}$ is the only characterized *ck1a* allele used in almost all genetic studies. It has been first described as a strong hypomorph or amorphic allele by the Treisman lab (Legent et al., 2012). $ck1a^{A}$ and $ck1a^{B}$ have been isolated in a large EMS screen by the Bellen lab (Haelterman et al., 2014), but both alleles have not been characterized so far. Based on the lamellipodia defects and impaired migratory behavior, we placed the mutations into the following allelic series: $ck1a^{[A]}$ > $ck1a^{8B12}$ > $ck1a^{G148S}$. We now included this information in the main text (see line 589-590).

-In Figure 2E they measure a bias angle?? What is this metric?? Its not an obvious migration parameter. Why not quantify numbers of hemocytes at the wound or something easier to interpret?

We apologize for our unclear description. Upon laser-induced wounding cells switch from a random migration mode to a directed migration mode. As suggested, we now counted the number of cells that reached the wound within the first 30 minutes after wounding (figure 3E). Cell number at the wound was normalized to the total amount of cells in a radius between 10 and 80 µm distance from the ablation site. We also measured the track speed mean of mutant cells compared to FRT control (figure 3F). Both, cell number at the wound and cell speed are significantly reduced in $ck1a^{A}$ as in mys^{1} and $wave^{\Delta 37}$ mutant cells. To further characterize the impaired migratory behavior of $ck1a^{A}$ mutant cells we still included the analysis of the bias angle (figure 3G and H). The bias angle describes the angle between a motion vector (a step of the cell) and the direction pointing toward the wound (see also Liepe et al., 2016). A bias angle close to 0° indicates the highest directionality to the wound, whereas cells with a value close to 180° move in opposite direction. For migrating wild type macrophages, the bias angles take values less than 80° (as shown in the new figure 3G and 3H as frequency distribution of the bias angle for each trajectory). By contrast, $ck1a^{A}$ mutant cells move with a bias angle between 80° and 180° (see new figure 3G and 3H). We also included the equation for calculating the bias angles in methods section. If required, we could further provide the R-script for calculating the bias angles.

-It is unclear what 2F is measuring. The angles don't look any different in the rose plot. Is there some metric that you could measure that can be statistically tested?

As mentioned before, we now also included a quantification of number of cells at the wound and track speed mean of mutant cells. To better describe changes in the migratory behavior we measured the bias angle that describes the angle between a motion vector (a step of the cell) and

© 2021. Published by The Company of Biologists under the terms of the Creative Commons Attribution License (https://creativecommons.org/licenses/by/4.0/).

the direction vector pointing toward the wound (see also Liepe et al., 2016). Observed changes in the bias angles were statistically tested (figure 3G). Figure 3H further shows the frequency distribution of the bias angle for each trajectory as a rose plot that is clearly different between wild type and mutant cells. For migrating wild type macrophages, the bias angles take values less than 80° (light blue bars). By contrast, *ck1a*^{A} mutant cells move with a bias angle between 80° and 180° (light red bars).

-Can 2I and 2J be quantified? One of their possible conclusions is that WAVE phosphorylation prevents its degradation and this result should be measured.

The reviewer is correct and we apologize for our unclear data presentation. Inhibition of CK1*a* using a specific kinase inhibitor (D4476) resulted in a significant reduction of WAVE protein levels in S2 cells (new figure 4C) The quantification of five independent experiments is shown in figure 4D. Consistently, we now provided new evidence that CK1*a* protects WAVE from degradation. RNAi-mediated CK1*a* depletion resulted in substantial reduction of WAVE protein level in macrophages (see figure 4E). The quantification of nine independent experiments is now shown in figure 4F.

As suggested by the reviewer, we also quantified the opposite experiment. However, the induced expression of full-length CK1*a* in stably transfected S2 cells did not increase WAVE protein abundance suggesting an already strong basal WAVE phosphorylation as previous described in mammals and in *Dictyostelium* (Kim et al., 2006; Ura et al., 2012). We decided to still include this experiment in supplementary figure 1A, and quantification of three independent experiments in figure 4H.

-The lethality experiments in 3B should be statistically compared.
As suggested we now statistically compared the rescue experiments (see new Figure 6B)

-In the text "Similarly, we still found significant defects in the migratory behavior of mutant macrophages…..(quantification in figure 3D)"…. Is this a typo? 3D is measuring circularity, not migration??

We apologize for this mistake. We now included both quantification of circularity (Figure 6D) and bias angle for changed migratory behavior (Figure 6E).

-Can 4A be quantified?

As suggested by the reviewer we now compared and quantified in more detail the overexpression of wild type WAVE-WT (Figure 6B, B'), phosphomimetic WAVE SD^{5x} (Figure 6C, C') and phosphomutant WAVE SA^{5x} (Figure 6D, D') in the posterior compartment of wing imaginal discs (New figure 6A-B). All three constructs were integrated into the same landing site (68E) to ensure an equal expression rate. We additionally used the expression of an EGFP as a negative control (Figure 6A, A') and *wave*RNAi transgene as a positive control (Figure 6E, E') for changes in F-actin level.
Our quantification confirmed that the overexpression of both wild type WAVE-WT and phosphomimetic WAVE SD^{5x} but not the phosphomutant WAVE SA^{5x} substantially induces F-actin in wing imaginal discs (see quantification in figure 6G). Interestingly, we found that WAVE SD^{5x} is more stable compared to WAVE WT and WAVE SA5x (see quantification in figure 6H).

-In the discussion the authors introduce additional data (4C and 4D). Please move to results.

As suggested we now included the CK2 data set into figure 1G-J.

-In the discussion they state that basal phosphorylation by CK protects WAVE against ubiquitin dependent degradation. Is there a reason they did not look at WAVE amount in hemocytes in vivo (or isolated from larvae) in the background of a CK mutant or CK RNAi (or even in whole animals)? This conclusion begs this experiment.

We agree and thank the reviewer for pointing out this. We now provided new evidence that CK1*a*

© 2021. Published by The Company of Biologists under the terms of the Creative Commons Attribution License (https://creativecommons.org/licenses/by/4.0/).

protects WAVE from degradation. RNAi induced CK1*α* depletion resulted in substantial reduction of WAVE protein level in macrophages (see figure 4E). The quantification of nine independent experiments is now shown in figure 4F.

Reviewer 2 Advance Summary and Potential Significance to Field: The Arp2/3 complex is one of the cell's major actin nucleators, shaping the cytoskeleton in many ways, including driving formation of lamellipodia in migrating cells. The WAVE complex is a critical regulator of Arp2/3. Here the authors provide new insights into the regulation of WAVE by phosphorylation, with a focus on casein kinase 1 alpha. They make superb use of the Drosophila hemocyte model, allowing them to combine RNAi and classical mutants and providing assays in vivo. Building on earlier work in vitro and in Dictyostelium, they find a striking effect of loss of casein kinase 1 alpha on both actin- based protrusions and motility. They identify potential CK1 phosphorylation sites in WAVE and via mutational analysis provide strong support for the idea that basal phosphorylation of sites in the VCA domain are important for WAVE stability and thus function.

The data are generally lovely, well quantified and the conclusions well supported. I think this will be of broad interest to cell and developmental biologists interested in cytoskeletal regulation. I did think there were places where the story was presented too rapidly and thus was hard to follow, and also think a few conclusions need to be further quantified or toned down. These issues are outlined below and should be straightforward to address.

We thank the reviewer for the very positive constructive comments.

Reviewer 2 Comments for the Author:

1. The manuscript starts with mention of a genetic screen of candidate genes using RNAi, and include a supplementary table mentioning the many candidates. This was described in a very cursory fashion. If this is because the screen itself will be published elsewhere, the authors should state that directly. Otherwise they should tell us more - for example was ck1 alpha the only hit? Also relevant – was CK2 included in the screen?

We apologize for our unclear description. As suggested by the reviewer, we further provided more information about the screen (see line 131- 140).

We also included more information about the candidates as follows: "Expression of most dsRNAs induced no defects in the cell morphology and lamellipodia formation. We identified the casein kinase 1α gene (Ck1α) as a candidate that most strongly affected lamellipodia formation and phenocopied *wave* depleted cells, characterized by a prominent reduced circularity index (Figure 1B; quantification in 1C). We only found a few more dsRNAs that only moderately affected cell shape, which will be published elsewhere (B. Nagel, unpublished results; see also supplementary table 1)."

Yes, indeed CK2 was also included in our initial RNAi screen which did not significantly affect lamellipodia formation but resulted in altered blood cell homeostasis (see also supplementary table 1). As suggested by reviewers, we now better introduce CK2 (see line 156-163 in the revised manuscript) and also included the CK2 mutant data set into figure 1G-J.

2. There were several places where data was referred to I could not find. The first was p. 5, line 12-14. Where are the data about "impaired spread morphology—if it is supposed to be apparent in the images they need to point this out, perhaps indicating particular cells.

We apologize for unclear description. Indeed, isolated *ck1a* mutant cells show impaired lamelipodia formation when plated ex vivo. We therefore changed this and now stated in the manuscript: "In *vivo*, *ck1a*^{8B12} and *ck1a*^{A} mutant macrophages migrate considerably slower" (line 182 in the manuscript).

The also should explicitly state that the effect on migration was only seen with 2 of the 3 alleles tested—this is OK as they have different lesions, but it needs to be stated.

We agree and included this information in the text (see line 142-152). The three different *ck1a*

© 2021. Published by The Company of Biologists under the terms of the Creative Commons Attribution License (https://creativecommons.org/licenses/by/4.0/).

alleles bear distinct missense mutations, at glycine 43 (*ck1a*^{8B12}), glycine 148 (*ck1a*^{G148S}, also known as *ck1a*^{B}) and lysine 141 (*ck1a*^{A}). The substitution of the lysine to methionine at position 141 (*ck1a*^{A}) is the only mutation that removes an H-bond affecting the active site of the CK1 (see three-dimensional structure, supplementary movie M1, dashed lines). By contrast, replacement of either glycine 43 (*ck1a*^{8B12}) or glycine 148 (*ck1a*^{B}) do not led to any obvious structural changes.

ck1a^{8B12} is the only characterized *ck1a* allele used in almost all genetic studies. It has been first described as a strong hypomorph or amorphic allele by the Treisman lab (Legent et al., 2012). *ck1a*^{A} and *ck1a*^{B} have been isolated in a large EMS screen by the Bellen lab (Haelterman et al., 2014), but both alleles have not been characterized so far. Based on the lamellipodia defects and impaired migratory behavior, we placed the mutations into the following allelic series: *ck1a* $^{[A]}$ > *ck1a* ^{8B12} > *ck1a* ^{G148S}. We now included the following information (see line 589-590).

3. The directional migration assay is an excellent one, but it is poorly described/displayed. Is the image of cells shown the initial image? Why do only a subset of the tracks start/end with a cell? Please indicate the start/end=direction of each trajectory, especially those that do not include an imaged cell.

We apologize for unclear description and further provide information about the wounding assay in the main text and in the figure legend as well. Cells were automatically tracked within the first 30 minutes post-wounding, the starting point of each trajectory is at the cell, the initial image is depicted at t=0 (Figure 3A-D). Macrophages migrate in three- dimension and migration behavior analysis is therefore based on three-dimensional position data of a cell over time. Thus, cells also migrate into the wound region at various later timepoints, after t=0.
As mentioned before, we now also included a quantification of number of cells at the wound and track speed mean of mutant cells. To better describe changes in the migratory behavior we measured the bias angle that describes the angle between a motion vector (a step of the cell) and the direction pointing toward the wound (Liepe et al., 2016; Weavers et al., 2016). Observed changes in the bias angles were statistically tested (figure 3G). Figure 3H further shows the frequency distribution of the bias angle for each trajectory as a rose plot that is clearly different between wild type and mutant cells. For migrating wild type macrophages, the bias angles take values less than 80° (light blue bars). By contrast, *ck1a*^{A} mutant cells move with a bias angle between 80° and 180° (light red bars).

They also state “A similar, but more severely impaired migratory behavior showed wave and ßPS-integrin mutant macrophages (Figure 2C, D; Moreira et al., 2013.” but the quantification in panel 2E does not show a difference in severity. Finally, are the wave and ßPS-integrin mutant macrophages from MARCM?

Yes indeed, to analyze embryonically lethal *mys*^{1} (ß-integrin) and *wave*$^{\Delta37}$ mutations in macrophages we also had to perform a MARCM analysis. The reviewer is correct. Based only on the bias angle quantification differences between *ck1a* and *wave*$^{\Delta37}$ are not obvious. However, quantification of additional parameters including the cell number at wound site and track speed mean shows that the migratory behavior *wave*$^{\Delta37}$ mutant macrophages is more severely impaired.

4. The phosphorylation assay used in Fig 2H should be briefly described in the results for those like me not familiar with this assay.

We now included a brief description of the assay in the results (line 218-224) and methods section (line 517-523).

5. The “slight increase” in WAVE levels after CK1alpha overexpression was not obvious— this statement should either be quantified or removed.

We quantified a possible effects CK1*a* overexpression on WAVE protein level (normalized with tubulin level) in seven independent experiments, but we found that there are no significant changes

© 2021. Published by The Company of Biologists under the terms of the Creative Commons Attribution License (https://creativecommons.org/licenses/by/4.0/).

upon CK1*α* overexpression. We now included this additional information in supplementary figure 1. However, we found that RNAi induced CK1*α* depletion results in a substantial reduction of WAVE protein level in macrophages (see figure 4E). The quantification of nine independent experiments is now shown in figure 4F. Thus, these data suggest that CK1*α* protects WAVE from degradation. The fact that overexpression CK1alpha did not further increase WAVE protein abundance suggest a prominent basal phosphorylation already stabilizes endogenous WAVE.

6. A reference should be given and a few more details provided documenting the specificity of the CK1alpha inhibitor D4476.

The CK1alpha inhibitor D4476 is a cell-permeable triaryl substituted imidazolo compound that acts as a potent and specific ATP-competitive inhibitor of CK1alpha. This has been proofed by the effect that the IC50 value for CK1 decreased progressively as the concentration of ATP was lowered (Rena, G., et al. 2004. EMBO Reports 5, 60). We included this information in the main text.

7. A little more clarity in the description of the "consensus motif" S/T-X-X-(X)-S/T would help—it isn't clear to me which of the S/T residues in the cluster they suggest match this motif. A minor point but it could be clearer. I get that it might have "moved" between flies and mammals.

We apologize for unclear description. We now included further information in the main text and marked the serines in red (figure 6A) that match the consensus sequence.

8. On page 7 they state: "Cells expressing the SA5x variant still showed a strongly reduced circularity compared to cells rescued either by wild type, SA3x or SD5x protein (quantification in figure 3D). Similarly, we still found significant defects in the migratory behavior of mutant macrophages expressing phosphomutant SA5x protein compared to wild type or phosphomimetic SD5x variant (quantification in figure 3D)." The former quantification is presented but the latter seems to be missing.

We apologize for this mistake. As mentioned before, we now included both quantification of circularity (Figure 6D) and bias angle for changed migratory behavior (Figure 6E).

In this same part of the manuscript, they should note and comment on the fact that the wildtype WAVE did not rescue the effect of ck1 RNAi (Fig. 3E)—this makes sense once you think about it but needs to be laid out more clearly.

We agreed and included a short notion/conclusion into the main text (line 275-276).

9. Are the actin images in Figure 4A scaled differently? It was odd that there was "more" actin in the control anterior compartment in one than the other.

As suggested by the first reviewer we now compared and quantified in more detail the overexpression of wild type WAVE-WT (Figure 6B, B'), phosphomimetic WAVE SD^{5x} (Figure 6C, C') and phosphomutant WAVE SA^{5x} (Figure 6D, D') in the posterior compartment of wing imaginal discs (new figure 6A-B). All three constructs were integrated into the same landing site (68E) to ensure an equal expression rate. We additionally used the expression of an EGFP as a negative control (Figure 6A, A') and *wave*RNAi transgene as a positive control (Figure 6E, E') for changes in F-actin level.

We found that WAVE SD5x is more stable compared to WAVE WT and WAVE SA5x (see quantification in figure 6H). By contrast, the protein levels of WAVE WT and WAVE SA5x are not significantly different. Despite the fact that phosphomimetic WAVE SD^{5x} is more stable than the wild type protein we found no increased activity (F-actin induction) between WAVE-WT and WAVE SD^{5x}.

10. The thing I found most confusing was the data in the rest of Figure 4, which is presented, for unexplained reasons, in a very rambling way and in the Discussion. This should probably be in the Results, likely as Figure 2, should be explained much better (only a handful of fly

© 2021. Published by The Company of Biologists under the terms of the Creative Commons Attribution License (https://creativecommons.org/licenses/by/4.0/).

immunologists would understand it) and needs to be coupled with mentioning whether CK2 came out of the original screen. The fact that they are very different enzymes (bottom of p 8) would be the preface for this.

We agree with the reviewer and we now included the CK2 data set into figure 1G-J and we also better described CK2 (see line 160-167).

Minor issues p. 5, line 13. Typo—"Figure 1H,I,J"
done

Reviewer 3 Advance Summary and Potential Significance to Field: Hirschhauser et al. investigated how the casein kinase 1α (CK1α) regulates Wave to control cell shape and migration. Using Drosophila macrophages as a model system, the authors first showed that genetic perturbation of ck1α impairs lamellipodial formation and migratory speed in primary culture condition and prepupal wound response in vivo. Subsequent biochemical analysis demonstrated that CK1α can interact with and phosphorylate WAVE, which protects WAVE from ubiquitin-mediated degradation. With further genetic analysis of putative phosphorylation sites in the N- and C-terminal regions of WAVE, the authors identified a cluster of C-terminal acidic residues critical for WAVE function in actin polymerization, lamellipodial formation, cell motility as well as embryonic development.

Overall, the authors made an important discovery of CK1α-mediated WAVE phosphorylation, which protects WAVE from degradation. Experiments are well designed and the data largely support the authors' conclusions. A major concern is the lack of in vivo evidence that supports phosphorylation-mediated WAVE stability.

Reviewer 3 Comments for the Author:
Specific comments:
1. Lack of in vivo demonstration of phosphorylation-mediated WAVE stability. One of the most exciting and novel findings of the manuscript is CK1α-mediated WAVE phosphorylation, resulting in the protection of WAVE from ubiquitin-mediated degradation. However, this is only shown in a biochemical assay using S2 cells. It is unclear if this also occurs in vivo.
In particular, the expression level of WAVE-SA5X appears to be similar to that of WAVE- SD5X, instead of lower (Fig 4A). Quantification is necessary here.

As suggested we now compared and quantified in more detail the overexpression of wild type WAVE-WT (Figure 6B, B'), phosphomimetic WAVE SD^{5x} (Figure 6C, C') and phosphomutant WAVE SA^{5x} (Figure 6D, D') in the posterior compartment of wing imaginal discs (new figure 6A-B). All three constructs were integrated into the same landing site (68E) to ensure an equal expression rate. We additionally used the expression of an EGFP as a negative control (Figure 6A, A') and *wave*RNAi transgene as a positive control (Figure 6E, E') for changes in F-actin level.

We found that WAVE SD5x is more stable compared to WAVE WT and WAVE SA5x (see quantification in figure 6H). By contrast, the protein levels of WAVE WT and WAVE SA5x are not significantly different. Despite the fact that phospho-mimetic WAVE SD^{5x} is more stable than the wild type protein we found no increased activity (F-actin induction) between WAVE-WT and WAVE SD^{5x}.

The authors may consider performing genetic interaction studies between proteasome components and WAVE rescue embryos and/or protein stability assays in embryos by western blots and/or immunofluorescence staining.

As suggested we have screened for proteasome components and tested various RNAi lines (see new figure 5). Ubiquitin-dependent degradation is a multi-step process that involves members of the cullin protein family as part of E3 ligase complexes, but also the 26S proteasome consisting of a 20S catalytic core and a 19S regulatory complex. Thus, we tested all 6 known cullins (Cul1-6) as well as four components of the 20S catalytic core (Prosβ5, Prosβ7) and the 19S regulatory complex (Rpn1, Rpn6). Indeed, RNAi mediated knockdown of Cul1 and 2, but not Cul3-6 rescued cell shape

© 2021. Published by The Company of Biologists under the terms of the Creative Commons Attribution License (https://creativecommons.org/licenses/by/4.0/).

defects of macrophages evoked by *ck1a* RNAi. Knockdown of any Cullin alone did not show a significant difference in cell shape. Likewise, when we affected the final step of proteasomal degradation by targeting either 20S catalytic core (Prosß5, Prosß7) or 19S regulatory complex we found a significant rescue of the spiky macrophage phenotype evoked by evoked by *ck1a* RNAi.

Thus, we provided further evidence that CK1α protects WAVE from ubiquitin-mediated proteasomal degradation.

2. Incomplete investigation and documentation of cell motility phenotype. The authors used circularity as a readout of lamellipodial formation for most genetic perturbation conditions. Track speed mean for cell motility should also be done for most genetic perturbation conditions. Currently, this assay is not done for the ck1α RNAi and WAVE phospho-mutant studies.

We now measured track speed means for the ck1α RNAi (see figure 2D), *ck1a* ^{L141M} (= *ck1α* ^{A}), *mys*^{1}, *wave*$^{\Delta 37}$ (figure 3 E) and included the bias angle for changes in the migratory behavior of macrophages rescued by WAVE phospho-mutant proteins (figure 6E)

Minor concerns: 1. Fig 1 and text: for all three ck1α alleles used in the study, it would be more consistent to use molecular lesion to mark the alleles (e.g. change [8B12] to [G43D], [A] to [L141M] in figures and texts).

As suggested we now changed all allele names, *ck1a*^{8B12} = *ck1a*^{G43D}, *ck1a*^{A} = *ck1a*^{L141M} and *ck1a*^{B} = *ck1a*^{G148S}.

2. ck1α[8B12] may not necessarily be an amorph, but instead a strong hypomorph, according to the previous study. Also, ck1α[A] is stronger than ck1α[8B12] in Fig 1. Does this mean that ck1α[A] could be an antimorphic allele?

The three different *ck1a* alleles bear distinct missense mutations, at glycine 43 (*ck1a*^{8B12}), glycine 148 (*ck1a*^{G148S}, also known as *ck1a*^{B}) and lysine 141 (*ck1a*^{A}). The substitution of the lysine to methionine at position 141 (*ck1a*^{A}) is the only mutation that removes an H- bond affecting the active site of the CK1 (see three-dimensional structure, supplementary movie M1, dashed lines). Given the strongest phenotype (cell morphology & cell motility) we believe that *ck1a*^{A} is an amorph, rather than an antimorph (= dominant-negative) or neomorph. The latter would imply possible phenotypic defects already in heterozygosity that we did not observe. By contrast, macrophages isolated from heterozygous females exhibit wild-type cell morphology.

ck1a^{8B12} is the only characterized *ck1a* allele used in almost all genetic studies. It has been first described indeed as a strong hypomorph by the Treisman lab (Legent et al., 2012). Replacement of either glycine 43 (*ck1a*^{8B12}) or glycine 148 (*ck1a*^{B}) do not led to any obvious structural changes (see supplementary movie M1). *ck1a*^{A} and *ck1a*^{B} have been isolated in a large EMS screen by the Bellen lab (Haelterman et al., 2014), but both alleles have not been characterized so far. Based on the lamellipodia defects and impaired migratory behavior, we placed the mutations into the following allelic series: *ck1a* $^{[A]}$ > *ck1a* ^{8B12} > *ck1a* ^{G148S}. We now included the following information (see line 589- 590).

3. Fig 1 does not show images for each genetic condition used in the quantification. Please include images for ck1α RNAi #1, wave RNAi, ck1α[8B12] and ck1α[G148S].

We now included the missing images into figure 1.

4. In Fig 2G-I, quantify all blots. Show all data points.

We now included the missing data points, now new figure 4D, F, H.

5. In Fig 2K, show all data points.

© 2021. Published by The Company of Biologists under the terms of the Creative Commons Attribution License (https://creativecommons.org/licenses/by/4.0/).

Done, now figure 4F.

6. In Fig 3B, why WAVE SD3X is more viable than WT?

We now quantified differences in rescue activity by different WAVE proteins (see figure 6B). We only found significant differences in rescue activity of the C-terminal phospho- mutant WAVE SA^{5x}. However, there are no significant differences between WAVE-WT, WAVE SA^{3x} and WAVE SD^{3x} (figure 6B). This is consistent with our rescue data for macrophages. Only phospho-mutant WAVE SA^{5x} failed to rescue defects in cell morphology (figure 6D), migratory speed (figure 6E) and behavior (figure 6F).

7. In Fig 3C and text, please clarify that these experiments are performed in wave[Δ37] mutant background and include SD3X image.

Yes, indeed all rescue experiments were done in wave[Δ37] mutant background. We also include SD3X image (now figure 6C).

Fig 3C is not referred in the text.
We changed this (now figure 6C).

8. In Fig 3D, include SD3X data.

We included SD3X data (now figure 6D).

9. It would be nice to have a model figure to illustrate the conclusion.

We included a model in figure 8.

Second decision letter

MS ID#: JOCES/2021/258891

MS TITLE: CK1α protects WAVE from degradation to regulate cell shape and motility in immune response

AUTHORS: Alexander Hirschhaeuser, Marianne van Cann, and Sven Bogdan
ARTICLE TYPE: Research Article

We have now reached a decision on the above manuscript.

To see the reviewers' reports and a copy of this decision letter, please go to: https://submit-jcs.biologists.org and click on the 'Manuscripts with Decisions' queue in the Author Area. (Corresponding author only has access to reviews.)

As you will see, the reviewers gave favourable reports but reviewer 3 raised some minor points that will require amendments to your manuscript. I hope that you will be able to carry these out because I would like to be able to accept your paper.

We are aware that you may be experiencing disruption to the normal running of your lab that makes experimental revisions challenging. If it would be helpful, we encourage you to contact us to discuss your revision in greater detail. Please send us a point-by-point response indicating where you are able to address concerns raised (either experimentally or by changes to the text) and where you will not be able to do so within the normal timeframe of a revision. We will then provide further guidance. Please also note that we are happy to extend revision timeframes as necessary.

© 2021. Published by The Company of Biologists under the terms of the Creative Commons Attribution License (https://creativecommons.org/licenses/by/4.0/).

Please ensure that you clearly highlight all changes made in the revised manuscript. Please avoid using 'Tracked changes' in Word files as these are lost in PDF conversion.

I should be grateful if you would also provide a point-by-point response detailing how you have dealt with the points raised by the reviewers in the 'Response to Reviewers' box. Please attend to all of the reviewers' comments. If you do not agree with any of their criticisms or suggestions please explain clearly why this is so.

Reviewer 1

Advance summary and potential significance to field

The additional data and clarification of experimental details requested in my previous review have been addressed by the authors. The paper is much improved.

Comments for the author

The additional data and clarification of experimental details requested in my previous review have been addressed by the authors. The paper is much improved.

Reviewer 2

Advance summary and potential significance to field

As I noted in my original review, the Arp2/3 complex is one of the cell's major actin nucleators, shaping the cytoskeleton in many ways, including driving formation of lamellipodia in migrating cells. The WAVE complex is a critical regulator of Arp2/3. Here the authors provide new insights into the regulation of WAVE by phosphorylation, with a focus on casein kinase 1 alpha. They make superb use of the Drosophila hemocyte model, allowing them to combine RNAi and classical mutants and providing assays in vivo.
Building on earlier work in vitro and in Dictyostelium, they find a striking effect of loss of casein kinase 1 alpha on both actin-based protrusions and motility. They identify potential CK1 phosphorylation sites in WAVE and via mutational analysis provide strong support for the idea that basal phosphorylation of sites in the VCA domain are important for WAVE stability and thus function. The data are lovely, well quantified and the conclusions well supported. The authors have fully addressed all of the issues I raised in initial review. I think this will be of broad interest to cell and developmental biologists interested in cytoskeletal regulation.

Comments for the author

Nice work!

Reviewer 3

Advance summary and potential significance to field

All of my questions have been addressed.

Comments for the author

I only have a few minor comments:
1. Fig 1H – show GFP negative cells to compare 2. Fig 1N – show all data points 3. Keep the order of data consistent between figures (Fig 1A-J vs. Fig 2)
for the ease of reading and comparing.
4. Besides the model presented, it would be nice to include an additional model illustrating ck1a-mediated wave stability in the cellular context, e.g.
including the molecular components and pathways.

© 2021. Published by The Company of Biologists under the terms of the Creative Commons Attribution License (https://creativecommons.org/licenses/by/4.0/).

Second revision

Author response to reviewers' comments

Reviewer 3 Comments for the author

I only have a few minor comments:

1. Fig 1H – show GFP negative cells to compare

We replaced the image in figure 1H with an eGFP negative cell as a control

2. Fig 1N – show all data points
We now included a new quantification showing all data points

3. Keep the order of data consistent between figures (Fig 1A-J vs. Fig 2)
for the ease of reading and comparing.

We now changed the order of data (different mutants) of figure 2 according to figure 1.

4. Besides the model presented, it would be nice to include an additional
model illustrating ck1a-mediated wave stability in the cellular context, e.g.
including the molecular components and pathways.

We now included molecular components of the proteasomal pathway found to be required for WAVE degradation (e.g. Cullin 1 and 2) in figure 8.

Third decision letter

MS ID#: JOCES/2021/258891

MS TITLE: CK1Î± protects WAVE from degradation to regulate cell shape and motility in immune response

AUTHORS: Alexander Hirschhaeuser, Marianne van Cann, and Sven Bogdan
ARTICLE TYPE: Research Article

I am happy to tell you that your manuscript has been accepted for publication in Journal of Cell Science, pending standard ethics checks.

© 2021. Published by The Company of Biologists under the terms of the Creative Commons Attribution License (https://creativecommons.org/licenses/by/4.0/).
